# Distinct Neural Properties in the Low-Frequency Region of the Chicken Cochlear Nucleus Magnocellularis

**DOI:** 10.1523/ENEURO.0016-17.2017

**Published:** 2017-04-11

**Authors:** Xiaoyu Wang, Hui Hong, David H. Brown, Jason Tait Sanchez, Yuan Wang

**Affiliations:** 1 Department of Biomedical Sciences, Florida State University College of Medicine, Tallahassee, FL 32306; 2 Program in Neuroscience, Florida State University, Tallahassee, FL 32306; 3 Roxelyn and Richard Pepper Department of Communication Sciences and Disorders, Northwestern University, Evanston, IL 60208; 4 Department of Psychology, Florida State University, Tallahassee, FL 32306; 5 Department of Neurobiology, Northwestern University, Evanston, IL 60208; 6 The Hugh Knowles Hearing Research Center, Northwestern University, Evanston, IL 60208

**Keywords:** low frequency processing, calcium binding protein, cholecystokinin, neural excitability, action potential

## Abstract

Topography in the avian cochlear nucleus magnocellularis (NM) is represented as gradually increasing characteristic frequency (CF) along the caudolateral-to-rostromedial axis. In this study, we characterized the organization and cell biophysics of the caudolateral NM (NMc) in chickens (*Gallus gallus*). Examination of cellular and dendritic architecture first revealed that NMc contains small neurons and extensive dendritic processes, in contrast to adendritic, large neurons located more rostromedially. Individual dye-filling study further demonstrated that NMc is divided into two subregions, with NMc2 neurons having larger and more complex dendritic fields than NMc1. Axonal tract tracing studies confirmed that NMc1 and NMc2 neurons receive afferent inputs from the auditory nerve and the superior olivary nucleus, similar to the adendritic NM. However, the auditory axons synapse with NMc neurons via small bouton-like terminals, unlike the large end bulb synapses on adendritic NM neurons. Immunocytochemistry demonstrated that most NMc2 neurons express cholecystokinin but not calretinin, distinct from NMc1 and adendritic NM neurons that are cholecystokinin negative and mostly calretinin positive. Finally, whole-cell current clamp recordings revealed that NMc neurons require significantly lower threshold current for action potential generation than adendritic NM neurons. Moreover, in contrast to adendritic NM neurons that generate a single-onset action potential, NMc neurons generate multiple action potentials to suprathreshold sustained depolarization. Taken together, our data indicate that NMc contains multiple neuron types that are structurally, connectively, molecularly, and physiologically different from traditionally defined NM neurons, emphasizing specialized neural properties for processing low-frequency sounds.

## Significance Statement

Low-frequency sounds are important for auditory perception and scene analysis, including speech recognition. Using an avian model sensitive to low-frequency hearing including infrasound, we characterized neuronal properties of a primary cochlear nucleus. We found that the neurons located at the low-frequency end of the tonotopic axis develop unique structural, synaptic, biochemical, and physiologic features, distinct from well-characterized neurons processing sounds of higher frequencies. These findings provide fundamental knowledge toward understanding the properties of low-frequency processing in the brain.

## Introduction

Topographic organization is a salient feature of sensory systems in the vertebrate brain (Moerel et al., 2014; Kaneko and Ye, 2015). In the auditory system, topography manifests as tonotopy, defined as the spatial representation of sound frequency in the brain. For optimally performing auditory tasks across sound frequencies, auditory neurons develop gradients of structural, synaptic, and intrinsic properties along the tonotopic axis. In the auditory brainstem, frequency-specific neuronal processing is tuned by tonotopic gradients of ion channel expression (von Hehn et al., 2004; Leao et al., 2006; Gazula et al., 2010), synaptic transmission and depression (Köppl, 1994; Fukui and Ohmori, 2004; Slee et al., 2010; Oline and Burger, 2014), and inhibitory kinetics (Tang et al., 2011; Wang et al., 2012).

In addition to the mechanisms associated with tonotopic gradients, studies in birds provide evidence that the auditory system may adopt a number of novel properties for processing low-frequency sounds. Birds can hear sound frequencies as low as 2–10 Hz, as demonstrated by behavior tests (Hill et al., 2014) and single-unit physiologic recordings (Warchol and Dallos, 1990). The avian nucleus magnocellularis (NM) is a primary cochlear nucleus and is analogous to the mammalian anteroventral cochlear nucleus (Ryugo and Parks, 2003). In chickens and owls, NM neurons display gradually increasing characteristic frequency (CF) from the caudolateral to rostromedial extent (Rubel and Parks, 1975; Takahashi and Konishi, 1988). NM neurons typically have few, short dendrites and receive excitatory inputs from the auditory nerve through large synapses on their cell bodies, the so-called end bulbs of Held (Ramón y Cajal, 1911; Boord and Rasmussen, 1963; Parks and Rubel, 1978; Rubel and Fritzsch, 2002). These structural specializations are thought to be important for processing temporally locked excitation and computing the location of sound source stimuli (Sullivan and Konishi, 1984; Oertel, 1985; Carr and Konishi, 1990; Warchol and Dallos, 1990; Trussell, 1999). Neurons located in the most caudolateral NM, however, appear devoid of end bulbs, favoring multiple traditional bouton synaptic specializations (Takahashi and Konishi, 1988; Köppl, 1994; Köppl and Carr, 1997; Fukui and Ohmori, 2004). NM neurons in this region have smaller cell bodies and possess extensive dendrites, in contrast to the typical adendritic morphology of higher CF neurons (Boord and Rasmussen, 1963). Regardless of these structural differences, *in vivo* recording studies demonstrate that NM neurons with low CFs perform temporal phase locking as accurate as, if not better than, neurons with higher CFs (Warchol and Dallos, 1990; Fukui et al., 2006, Oline et al., 2016). These studies suggest that the auditory system may develop distinct neuronal properties for similar function in temporal processing at different frequencies.

The current study provides a systematic characterization of the organization, connectivity, and neural properties of the caudolateral NM distinct from higher-frequency regions of the nucleus. Using a combination of neuroanatomical and physiologic approaches, we identify two caudolateral NM subregions, NMc1 and NMc2. NMc1 and NMc2 are distinct from adendritic NM neurons in the expression of a neuropeptide and calcium-binding proteins, in addition to their extensive dendritic development and bouton-like synapses with the auditory axons. Importantly, NMc1 and NMc2 neurons display the ability of generating multiple action potentials following sustained current injections and show heterogeneity in their spiking activity, features not found in adendritic NM neurons.

## Materials and Methods

### Animals

White leghorn chicken embryos and hatchlings (*Gallus gallus*) of either sex were used. Chickens take ∼21 days to hatch. We used chickens from late embryonic stage at embryonic day 19 (E19) up to 2 wk posthatch (P14). At this age range, near-mature hearing ability is established (Saunders et al., 1974; Rebillard and Rubel, 1981), and NM neurons have obtained mature-like morphology and physiology (Jhaveri and Morest, 1982; Burger et al., 2005b; Sanchez et al., 2010, 2012a, 2015a). Eggs for anatomic studies were obtained from Charles River Laboratories and incubated in a Florida State University vivarium. Eggs for electrophysiological studies were obtained from Sunnyside Farms and incubated in the central auditory physiology laboratory at Northwestern University. All procedures were approved by the Florida State University and Northwestern University Institutional Animal Care and Use Committees and conducted in accordance with the National Institutes of Health Guide for the Care and Use of Laboratory Animals.

### Immunohistochemistry

Chicken hatchlings (P2–P14; *n* = 21) were transcardially perfused with 0.9% saline followed by 4% paraformaldehyde in 0.1 m phosphate buffer (PB). The brains were removed from the skull, postfixed overnight in 4% paraformaldehyde, and transferred to 30% sucrose in PB with 0.02% sodium azide. Brains were then sectioned in the coronal plane at 30 μm on a freezing sliding microtome. Each section was collected in 0.01 m PBS with 0.02% sodium azide. Alternate serial sections were immunohistochemically stained for primary antibodies listed in Table 1, following the protocol described previously (Wang et al., 2009). Briefly, free-floating sections were incubated with primary antibody solutions diluted in PBS with 0.3% Triton X-100 overnight at 4°C, followed by Alexa Fluor secondary antibodies (Invitrogen) at either 1:200 for 4 h at room temperature or 1:1000 overnight at 4°C. Some sections were counterstained with NeuroTrace (Invitrogen), a fluorescent Nissl stain, at 1:1000 incubated together with secondary antibodies. Sections were then mounted on gelatin-coated slides and coverslipped with Fluoromount-G mounting medium (Southern Biotechnology).

**Table 1.** Primary antibodies used for immunostaining

Antibody	Manufacturer	RRID	Host species	Working concentration
Calretinin	Millipore; AB5054	AB_2068506	Rabbit	1:5000
CCK	Sigma; C2581	AB_258806	Rabbit	1:2000
CTB	List Biological Lab; 703	AB_10013220	Goat	1:12,000
Gephyrin	Synaptic Systems; mAb7a	AB_2314591	Mouse	1:500
MAP2	Millipore; MAB3418	AB_94856	Mouse	1:1000
Parvalbumin	Sigma; P3088	AB_477329	Mouse	1:5000
SNAP25	Millipore; MAB331	AB_94805	Mouse	1:1000

CCK, Cholecystokinin; CTB, Cholera toxin B; MAP2, microtubule-associated protein 2; SNAP25, synaptosome associated protein 25.

For peroxidase staining with a single antibody, after primary antibody incubation, sections were incubated in a biotinylated IgG antibody (1:200; Vector Laboratories) diluted in PBS with 0.3% Triton X-100 for 1 h at room temperature. After washing in PBS, sections were incubated in avidin–biotin–peroxidase complex solution (ABC Elite kit; Vector Laboratories) diluted 1:100 in PBS with 0.3% Triton X-100 for 1 h at room temperature. Sections were then washed in PBS and incubated for 3–5 min in 0.045% 3-3-diaminobenzidine (Sigma-Aldrich) with 0.03% hydrogen peroxide in PB. Sections were mounted on gelatin-coated slides and dehydrated, cleared, and coverslipped with Permount mounting medium (Fisher Scientific).

### Quantitative analysis of NM cell body size

**Figure 1.**
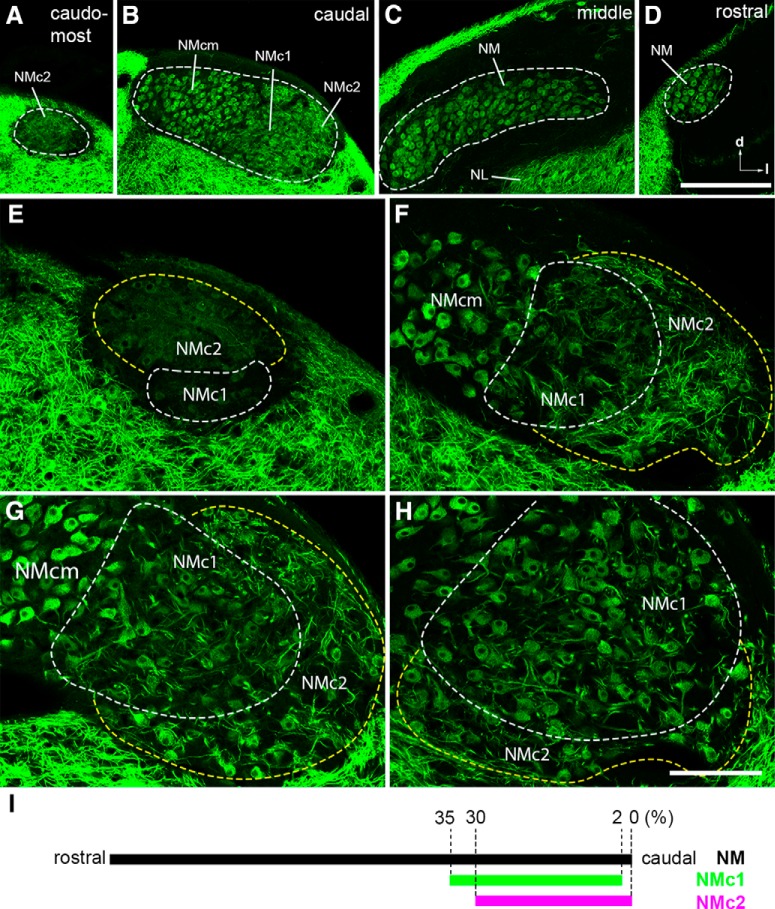
Three subdivisions of the caudal NM revealed by MAP2 immunoreactivity. ***A–D***, Low-magnification images taken from the caudomost (***A***), caudal (***B***), middle (***C***), and rostral (***D***) regions of NM at the coronal plane. To visualize MAP2 staining in NM, the images were saturated in the surrounding tissues that are stained more strongly for MAP2 immunoreactivity than NM and NL. Dashed lines outline the border of NM. ***E–H***, High-magnification images of the caudolateral NM. Dashed white and yellow lines outline the border of NMc1 and NMc2, respectively. Images in ***E*** and ***F*** were taken from the level between ***A*** and ***B***. Image in ***G*** was taken from the same section in ***B***, whereas the image in ***H*** is at a level slightly rostral to ***B*** and ***G***. Note distinct staining pattern of MAP2 between NMcm, NMc1, and NMc2. For each image, right is lateral and up is dorsal. ***I***, The relative location of NMc1 and NMc2 along the caudal–rostral axis in series coronal sections through NM. Abbreviations: l, lateral; d, dorsal; NM, nucleus magnocellularis; NMcm, caudomedial NM; NMc1, caudolateral NM subregion 1; NMc2, caudolateral NM subregion 2. Scale bars = 200 μm in ***D*** (applies to ***A–D***) and 100 µm in ***H*** (applies to ***E–H***).

This analysis was performed on P12–P14 chickens (*n* = 4). For each animal, every fourth section containing NM was triple labeled for MAP2 and calretinin immunoreactivity as well as for NeuroTrace. This generates three to four coronal sections from each animal containing the three NM subregions defined in the current study, NMcm, NMc1, and NMc2 (see Fig. 1 and Results for definition of the subregions). All sections were imaged at single focal plane with a 63× objective lens attached to a Zeiss LSM 880 confocal microscope. All images from the same animal were captured using the same imaging parameters. The criteria for including a cell in the subsequent analysis were as follows: (1) MAP2 positive, (2) able to be unambiguously grouped into either NMcm, NMc1, or NMc2 based on MAP2 and calretinin staining, and (3) with a well-defined cell boundary and an identifiable nucleus in NeuroTrace staining. Cross-sectional somatic area of each selected neuron was measured from NeuroTrace staining using ImageJ (National Institutes of Health).

**Figure 2.**
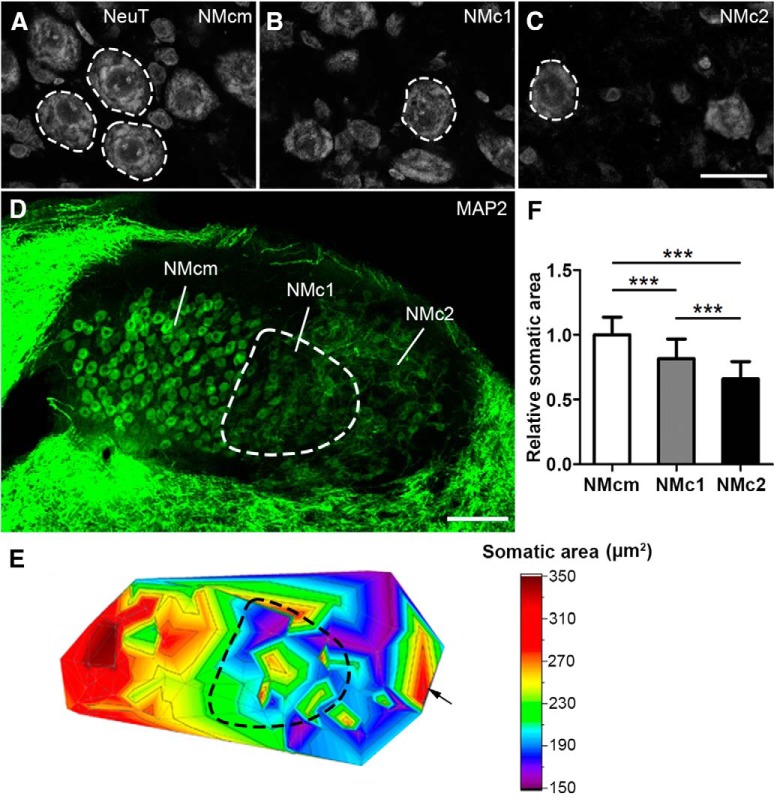
Comparison of neuronal cell body size in NMcm, NMc1, and NMc2. ***A–C***, NeuroTrace stain in NMcm (***A***), NMc1 (***B***), and NMc2 (***C***) on coronal sections. White dashed circles illustrate examples of measured neurons. ***D***, Low-power image of MAP2 immunostaining on the section containing the three subregions. The NMc1 is outlined with dashed line. ***E***, Projection of 3D color map surface plot representing the somatic area of NM neurons in relation to their location on the section shown in ***D***. Warm colors represent larger cells. The NMc1 in D is indicated accordingly by dashed line. Arrow indicates a group of large cells along the lateral edge of NMc2. ***F***, Bar chart of the cross-sectional relative somatic areas in NMcm, NMc1, and NMc2. ***, significant difference (*P <* 0.001). Data are presented as mean ± SD. Abbreviations: see Fig. 1. Scale bar = 20 μm in ***C*** (applies to ***A–C***) and 100 μm in ***D***.

To map the somatic area in relationship to the location of each measured neuron, a vertical line was drawn at the location of the most medially located NM neuron measured in one section and served as the *y*-axis. A horizontal line was drawn at the location of the most ventrally located NM neuron measured in the same section and served as the *x*-axis. The location of each measured neuron was then calculated according to these coordinates using ImageJ. Somatic areas were presented as the *z*-axis in relationship to the *xy* locations as a projection of 3D color map (see Fig. 2*E*), created using OriginPro (OriginLab).

For statistical analyses of grouped data across animals, the somatic area of each measured neuron was normalized to the average somatic area of all measured neurons in NMcm of the same animal. The normalized somatic areas of all measured neurons from all three animals were grouped for each NM subregion and compared between NMcm, NMc1, and NMc2. Significance was determined by one-way ANOVA and unpaired *t* test using the Prism version 5 software package (GraphPad). *P* < 0.05 was considered statistically significant. All data are shown as mean ± SD in the text and figures.

### Cell counting of NM neurons expressing cholecystokinin, calretinin, or parvalbumin

This analysis was performed on P12–P14 chickens for calretinin (*n* = 4) and P6 chickens for cholecystokinin (CCK; *n* = 3) and parvalbumin (*n* = 3). For each animal, every fourth coronal section containing the NM was triple-labeled for MAP2 immunoreactivity, NeuroTrace, and CCK, calretinin, or parvalbumin immunoreactivity. The caudomost three sections contain the main body of NMc1 and NMc2 as well as a substantial portion of NMcm were used for cell counting. MAP2 and NeuroTrace staining were used to identify NM subregions and visualize neuronal cell bodies of all NM neurons. Average optic density of somatic CCK, calretinin, or parvalbumin immunoreactivity was measured using ImageJ. For calretinin and parvalbumin staining, neurons whose staining intensity was 2 SDs above the mean background level were considered as positive neurons. Here the background is defined as a region in the tissue without identifiable cell bodies or processes present. For CCK staining, neurons whose staining intensity was 2 SD above the mean level of NMcm neurons were considered as positive neurons (see Results for the rationale). CCK-, calretinin-, or parvalbumin-positive neurons were counted in each NM subregion using the cell counter function of ImageJ. The percentage of CCK-, calretinin-, or parvalbumin-positive neurons was compared with the total MAP2-labeled neuronal number. Significance was analyzed by χ^2^ test using SPSS Statistics version 19.0 (IBM). As a second type of analysis, mean grayscale of CCK immunoreactivity was analyzed by one-way ANOVA and Bonferroni’s multiple comparison posttest using Graphpad Prism. *P* < 0.05 was considered statistically significant. All data are shown as mean ± SD in the text and figures.

### *In vitro* single-cell filling in brainstem slices

#### Slice preparation

Chicken brainstems at E19 (*n* = 3) were prepared as previously described (Hong et al., 2016). Briefly, the brainstems were dissected out in ice-cold oxygenated artificial CSF (ACSF) at pH 7.2–7.4 containing the following (in mM): 130 NaCl, 2.5 KCl, 1.25 NaH_2_PO_4_, 26 NaHCO_3_, 1 MgCl_2_, 3 CaCl_2_, and 10 glucose. ACSF was continuously bubbled with a mixture of 95% O_2_/5% CO_2_ for dissection and incubation. Coronal sections (300 µm) containing the caudal NM were prepared with a vibratome (Pelco easiSlicer, Ted Pella) and collected into a slice incubation chamber. Slices were incubated at 37°C for 40 min before switching to room temperature for cell filling.

#### Cell filling

Neurons in the caudal NM were individually dye-filled using electroporation (Wang and Rubel, 2012). Briefly, a glass pipette filled with fixable Alexa Fluor 568 dextran (Invitrogen) was driven to approach an identifiable cell body under a Zeiss V16 stereo-fluorescence microscope. The dye was introduced into the cell by a positive voltage (15–30 V, 20-ms pulse duration, 20 pulses/s, 1–5 s). After electroporation, slices were incubated for another 1–2 min to allow dye diffusion to distal dendrites. Slices were then fixed with 4% paraformaldehyde for 15 min at room temperature. After washing with PBS, sections were counterstained with NeuroTrace and mounted on noncoated slides with Fluomount mounting medium. To reduce tissue shrinkage, a nail polish spot was made at each corner of the coverslip to increase the space between the slide and coverslip.

#### Dendritic structural analyses

Using the Zeiss LSM 880 confocal microscope, image stacks of each dye-filled neuron were collected with 63× oil-immersion lens at a resolution of 0.26 µm per pixel at *xy* dimensions and with a *z* interval of 0.4 µm. These imaging settings provide sufficient resolution for accurate reconstruction and identification of distal ending morphology. Neurons with the entire dendritic arborization contained within one slice were used for subsequent 3D reconstruction. Neurons with dendrites extending outside of the slice were excluded from this analysis. Image stacks were converted into a series of TIFF images in Zeiss Zen Blue software and then imported to Neurolucida (version 9.03; MBF Bioscience). The entire dendritic arborization was traced with lines through the middle of each branch, as previously described (Wang and Rubel, 2012). Based on this reconstruction, the number of primary dendritic trees and the total dendritic branch length (TDBL) were measured using Neurolucida Explorer (version 9.03; MBF Bioscience). TDBL was calculated as the sum of the length of all dendritic branches of a neuron. No tissue shrinkage correction was applied.

After imaging, the coverslips were washed with PBS, and the slices were removed from the slides and resectioned at 30 μm. Double immunostaining against MAP2 and calretinin was then performed to determine the location of the filled neurons in NM subregions. TDBL and the number of primary trees were compared between neurons in different NM subregions, using one-way ANOVA with unpaired *t* test with Prism. Welch’s correction was used when the variances were not equal. *P* < 0.05 was considered statistically significant. All data are shown as mean ± SD in the text and figures.

### *In vitro* injection into the eighth nerve

E19 chicken embryos (*n* = 6) were used for this experiment. Brainstem blocks 3–4 mm thick attached with the surrounding skull were prepared in oxygenated ACSF to expose the eighth cranial nerve. The eighth nerve consists of an auditory branch and two vestibular branches (D’Amico-Martel, 1982; D’Amico-Martel and Noden, 1983; Kaiser and Manley, 1996). Before injection, the surface of the nerve branches was briefly dried with low-pressure carbogen (95% O_2_/5% CO_2_) blown through a syringe. We then injected the axonal bundle by using a metal needle whose tip was covered with dextran Alexa Fluor 488, 10,000-MW crystals (Invitrogen). For each animal, we made one injection into the auditory branch on one side of the brain and a second injection into the larger bundle of vestibular branches that is located rostral and ventral to the auditory branch on the other side of the brain. After injection, the brainstem chunks were dissected out from the skull with special care to preserve the eighth nerve. The brainstems with attached nerve were then incubated in oxygenated ACSF for another 6 h at room temperature before immersion fixation with 4% paraformaldehyde overnight at 4°C. After cryoprotection with sucrose, brainstems were sectioned at 30 µm as described above, immunostained or counterstained with NeuroTrace, and mounted on gelatin-coated slides for subsequent imaging.

### *In vivo* injection into the superior olivary nucleus

On the day of hatching (P0), chickens (*n* = 3) were anesthetized with a ketamine (60 mg/kg) and xylazine (8 mg/kg) cocktail administered intramuscularly. Feathers were plucked from the head, and an incision was made to expose the dorsal skull. The animal was secured in a custom stereotaxic head holder designed to allow calibrated rotation of the head. A 0.5-mm hole was drilled 2.0 mm lateral to midline and 0.3 mm caudal to the suture joining the frontal and parietal skull. To target the superior olivary nucleus (SON), a glass micropipette (tip diameter 40–60 μm) was filled with 1% cholera toxin B (CTB; List Laboratories) and advanced into the brain at a 6°–10° rotation in the rostrocaudal axis and a 4° rotation in the mediolateral axis, to a depth of ∼9 mm. Tracer was pressure ejected with a Picospritzer II (General Valve Corp.) using 10- to 50-ms pulses at 20 psi. The micropipette was retracted, the hole covered with bone wax, and the incision was closed. After survival of 3–6 d, chicks were deeply anesthetized with sodium pentobarbital and transcardially perfused with saline followed by 4% paraformaldehyde. Brains were extracted from the skull and postfixed for 24 h in 30% sucrose in paraformaldehyde until they sank. The brains were then sectioned and immunostained for CTB, calretinin, and CCK (Table 1). Calretinin and CCK serve as biomarkers for identifying NMc1 or NMc2 (see Results).

### Imaging for illustration

Images for illustration were captured with either a Zeiss M2 microscope for bright-field and epifluorescent images or a Zeiss LSM 880 confocal microscope. Epifluorescent images taken with the M2 microscope were treated with the Zeiss apotome, an optical sectioning approach using structured illumination for reducing out-of-focus information in epifluorescent images (Neil et al., 1997, 2000). Photomontages were applied in Zeiss Zen Blue software. Image brightness, gamma, and contrast adjustments were performed in Photoshop (Adobe Systems). All adjustments were applied equally to all images of the same set of staining from the same animal unless stated otherwise.

### *In vitro* electrophysiology in brainstem slices

#### Slice preparation

Acute brainstem slices were prepared from chicken embryos from E20–E21, as previously described (Sanchez et al., 2010). Briefly, the brainstem was dissected and isolated in oxygenated low-Ca^2+^ high-Mg^2+^ modified ACSF containing the following (in mm): 130 NaCl, 2.5 KCl, 1.25 NaH_2_PO_4_, 26 NaHCO_3_, 3 MgCl_2_, 1 CaCl_2_, and 10 glucose. ACSF was continuously bubbled throughout the experiments with a mixture of 95% O_2_/5% CO_2_ (pH 7.4, 295–310 mOsm/l). The brainstem was blocked coronally, affixed to the stage of a vibratome slicing chamber (Ted Pella), and submerged in ACSF. Bilaterally symmetrical coronal slices were made (200 µm thick), and approximately seven slices containing NM were taken from caudal to rostral, roughly representing the low-to-high frequency regions, respectively. The caudomost two to three slices were used in the current study.

Slices were collected in a custom holding chamber and allowed to equilibrate for 1 h at ∼22°C in normal ACSF containing the following (in mm): 130 NaCl, 2.5 KCl, 1.25 NaH_2_PO_4_, 26 NaHCO_3_, 1 MgCl_2_, 3 CaCl_2_, and 10 glucose. Normal ACSF was continuously bubbled with a mixture of 95% O_2_/5% CO_2_ (pH 7.4, 295–310 mOsm/l). Slices were transferred to a recording chamber mounted on an Olympus BX51W1 microscope for electrophysiological experiments. The microscope was equipped with a CCD camera, 60× water-immersion objective, and infrared differential interference contrast optics. The recording chamber was superfused continuously (Welco) at room temperature (monitored continuously at ∼22°C, Warner Instruments) in normal oxygenated ACSF at a rate of 1.5–2 ml/min.

#### Whole-cell electrophysiology

Current-clamp experiments were performed using an Axon Multiclamp 700B amplifier (Molecular Devices). Patch pipettes were pulled to a tip diameter of 1–2 μm using a P-97 flaming/brown micropipette puller (Sutter Instrument) and had resistances ranging from 3 to 6 MΩ. The internal solution of patch pipettes was potassium based and contained the following (in mm): 105 K-gluconate, 35 KCl, 1 MgCl_2_, 10 HEPES-K^+^, 5 EGTA, 4 4-ATP-Mg^2+^, and 0.3 4-Tris2GTP, pH adjusted to 7.3–7.4 with KOH. The junction potential was approximately –10 mV and was not corrected for current-clamp data reported in this study.

After a GΩ seal was attained, membrane patches were ruptured, and neurons were first held in the voltage clamp mode of whole-cell configuration. A small hyperpolarizing (–1 mV, 30 ms) voltage command was presented to monitor whole-cell parameters (i.e., cell membrane capacitance, series resistance, and input resistance). NM neurons were included in the data analysis only if they had series resistances <15 MΩ. Afterward, we switched to current clamp mode at I = 0 for further recordings. Raw data were low-pass filtered at 2 or 5 kHz and digitized at 20 or 50 kHz using a Digidata 1440A (Molecular Devices).

Pipettes were visually guided to the NMc, where neurons were identified and distinguished from surrounding tissue based on cell morphology and location of the nucleus within the slice. In a subset of experiments (*n* = 7), 0.1% neurobiotin was added to the pipette solution. Whole-cell patch-clamp recordings were conducted for ∼5 min, and tissue was immediately fixed in 4% paraformaldehyde. The location and morphology of NMc1/NMc2 neurons were confirmed using confocal microscopy (see Fig. 9*A*).

All experiments were conducted in the presence of a GABA_A_-R antagonist picrotoxin (PTX, 100 μm). Synaptic glutamate transmission was continuously blocked using DL-2-amino-5-phosphonopentanoic acid (DL-APV, 100 μm, an NMDA-R receptor antagonist) and 6-cyano-7-nitroquinoxaline-2,3-dione (CNQX, 20 μm, an AMPA-R receptor antagonist). Passive membrane properties and action potential (AP) properties were recorded and characterized by using different current clamp protocols. To measure the passive membrane properties, a small hyperpolarizing current was injected into the soma (–10 pA; Franzen et al., 2015, Hong et al., 2016). This paradigm minimizes the recruitment of voltage-dependent ion channels that are not active at or near rest. Membrane voltages used for data analysis were averaged over 30 repetitive trials and calculated by fitting a single exponential to the first 30-ms time window after the hyperpolarizing current injection. The membrane input resistance (R_M_) was obtained by dividing the calculated steady-state membrane voltage by the injected current. The time constant of the membrane voltage (τ_M_) was quantified by fitting a single exponential as described above, and membrane capacitance (C_M_) was calculated as C_M_ = τ_M_/R_M_. AP threshold current is defined as the minimum amount of current required for neurons to generate an AP ∼50% of the time across 30 repetitive stimulations (interpulse stimulus intervals = 2 s). Once AP threshold current was obtained, a sustained current command (duration = 100 ms) was injected into the soma at 25% above the measured threshold current for each neuron. APs evoked by this current command were used to characterize AP properties. Each AP property was measured and averaged over 30 repetitive trials.

#### Data analysis

Recording protocols were written and run using Clampex acquisition and Clampfit analysis software (version 10.3; Molecular Devices). Statistical analyses and graphing protocols were performed using Prism (version 6.07) and Matlab (version R2014b; The Math Works). Correlation analyses were conducted to explore the relationships between AP properties and reported as Pearson product-moment correlation (*r*). A linear regression was fitted to scatter plots. The standard for a significant correlation was defined as *p* < 0.05. All data are shown as mean ± 1 SD in the table and text.

#### Reagents

All bath-applied drugs were allowed to perfuse through the recording chamber for ∼10 min before subsequent recordings. DL-APV, CNQX, and all other salts and chemicals were obtained from Sigma-Aldrich. PTX were obtained from Tocris. Neurobiotin was obtained from Vector Laboratories.

## Results

### The caudal NM contains two neuronal groups with dendrites, NMc1 and NMc2

The classic NM neurons are characterized by a round, bald soma with no or only one or two short dendrites (Ramón and Cajal, 1911; Jhaveri and Morest, 1982). This adendritic morphology, however, is not common to neurons in the caudal NM. To visualize neuronal dendrites, we first examined the distribution pattern of MAP2 immunoreactivity, which labels all neuronal somata and dendrites (Fig. 1). As expected, the middle and rostral portions of NM display strong somatic staining without substantial dendritic structure (Fig. 1*C*, *D*). At the caudal level, this staining pattern is restricted to the medial region, referred to as the caudomedial NM (NMcm) for subsequent description (Fig. 1*B*). In contrast, extensive dendritic staining is seen in the lateral region of the caudal NM (Fig. 1*B*) as well as the most caudal pole of the NM (Fig. 1*A*). High-magnification observations further revealed that the caudolateral NM containing neuronal dendrites is divided into two subregions, here named NMc1 and NMc2 (Fig. 1*E–H*). NMc1 is located immediately adjacent to adendritic neurons in NMcm. NMc2 surrounds NMc1 caudally and laterally and occupies the most caudal pole of NM. Compared with NMc1, NMc2 shows longer MAP2-stained dendritic branches and overall higher intensity of MAP2 immunostaining. We further mapped the relative location of NMc1 and NMc2 along the caudal–rostral axis in series coronal sections through NM (Fig. 1I). NMc1 and NMc2 are found in the most caudal one-third of the entire NM. NMc1 and NMcm usually disappear from the most caudal coronal section, corresponding to only approximately 2% of the caudal–rostral axis.

MAP2-stained neuronal cell bodies in NMc1 and NMc2 appear smaller in size than those in NMcm (Fig. 1*G*). To quantitatively confirm this observation, we measured cross-sectional somatic area of NM neurons from sections stained with NeuroTrace, a fluorescent Nissl stain, and MAP2 (Fig. 2*A–C*). We first mapped the somatic area to the location of measured cells in individual coronal sections as a projection of 3D heat map (Fig. 2*D*, *E*). This map clearly shows larger cell body sizes in NMcm (warm colors in Fig. 2*E*) and smaller cell body sizes in NMc1 and NMc2 (cold colors). This general distribution pattern was found in all animals examined. Notably, although cells of different sizes are intermingled in both NMc1 and NMc2, the majority of cells with the smallest cell body sizes (blue color) are located in NMc2. Interestingly, NMc2 also contains a small number of neurons with relatively large cell body sizes as shown in warm colors. These large cells are found widely in NMc2, although they tend to cluster in the most lateral region of NMc2 in some animals (arrow in Fig. 2*E*). Statistical analysis on population data across sections and animals further confirmed that the somatic area in NMcm (*n* = 63 cells from three animals) is significantly larger than that in NMc1 (*n* = 62 cells from three animals, *p* < 0.0001) and NMc2 (*n* = 63 cells from three animals, *p* < 0.0001; Fig. 2*F*). In addition, the somatic area of the neurons in NMc1 is significantly larger than that in NMc2 (*p* < 0.0001).

**Figure 3.**
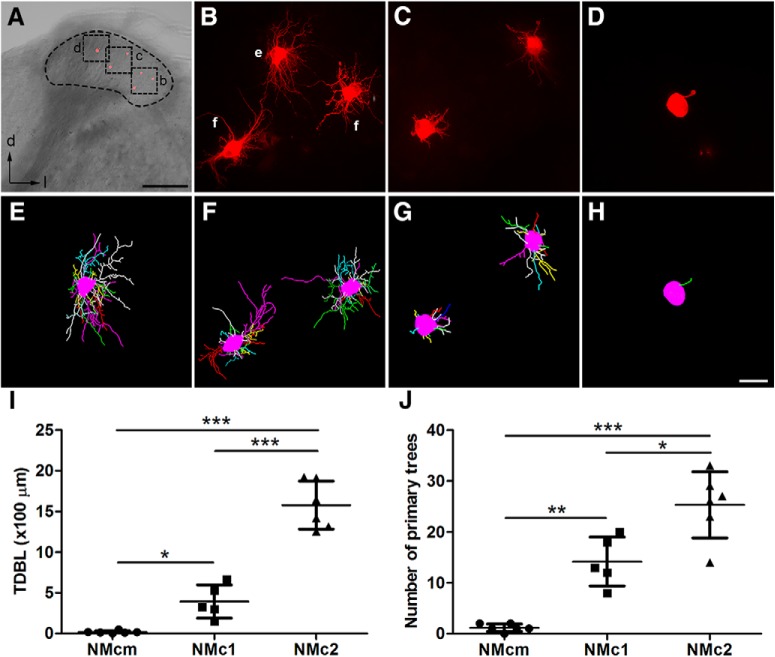
Single-cell dye-filling shows different dendritic morphology in NMcm, NMc1, and NMc2. ***A***, An example slice containing several filled neurons in different subregions along the lateral-to-medial axis. The black dashed circle outlines NM. Cell bodies of the filled neurons (red) are evident in this low-magnification image. ***B–D***, Higher magnification of the boxes in ***A*** showing maximum *z*-projection of filled neurons in NMc2 (***B***), NMc1 (***C***), and NMcm (***D***). ***E***, ***F***, 3D reconstruction of the filled neurons in ***B***. ***G***, 3D reconstruction of the filled neurons in ***C***. ***H***, 3D reconstruction of the filled neuron in ***D***. ***I***, Quantitative analysis of the total dendritic branch length. ***J***, Quantitative analysis of the number of primary trees. ***, *P <* 0.001; **, *P <* 0.01; *, *P <* 0.05. Data are presented as mean ± SD. Abbreviations: see Fig. 1. Scale bars = 200 μm in ***A*** and 20 µm in ***H*** (applies to ***B–H***).

To further examine the dendritic morphology of individual neurons in NMc1 and NMc2, we filled individual cells with a fluorescent dye in the caudal NM. Fig. 3*A* shows a coronal brainstem slice containing NMcm, NMc1, and NMc2 located from medial to lateral. Consistent with the observations from MAP2 staining, filled neurons in the most medial NM have either no dendrites or only one short dendrite (Fig. 3*D*, *H*), whereas neurons located more laterally show extensive dendrites. In particular, filled neurons in the most lateral portion where NMc2 is located show notably more dendritic branches (Fig. 3*B*, *E*, *F*) than the neurons in NMc1 (Fig. 3*C*, *G*). We further quantified dendritic structural properties based on 3D reconstruction of the dendritic arborization of individual filled neurons. As expected, the TDBL of NMcm neurons (18 ± 17 µm; *n* = 6 cells from three animals) is significantly smaller than that of NMc1 (392 ± 204 µm; *n* = 5 cells from three animals; *p* = 0.0148) and NMc2 neurons (1577 ± 294 µm; *n* = 6 cells from three animals; *p* < 0.0001; Fig. 3*I*). In addition, the TDBL of NMc2 neurons is significantly larger than that of NMc1 neurons (*p* < 0.0001). Similarly, NMcm neurons have fewer than two primary dendrites on average (1.2 ± 0.8), significantly less than NMc2 (25.3 ± 6.5; *p* = 0.0003) and NMc1 neurons (14.2 ± 4.8; *p* = 0.0039; Fig. 3*J*). NMc2 neurons have significantly more primary dendrites than NMc1 neurons (*p* = 0.011). Importantly, increases in TDBL from NMcm to NMc1 and from NMc1 to NMc2 are rather robust; there is no overlap of the TDBL ranges between the three regions. Together, these data demonstrate significantly increasing dendritic size and complexity from NMcm to NMc1 and from NMc1 to NMc2.

### NMc2 is distinct from other NM portions in CCK distribution

**Figure 4.**
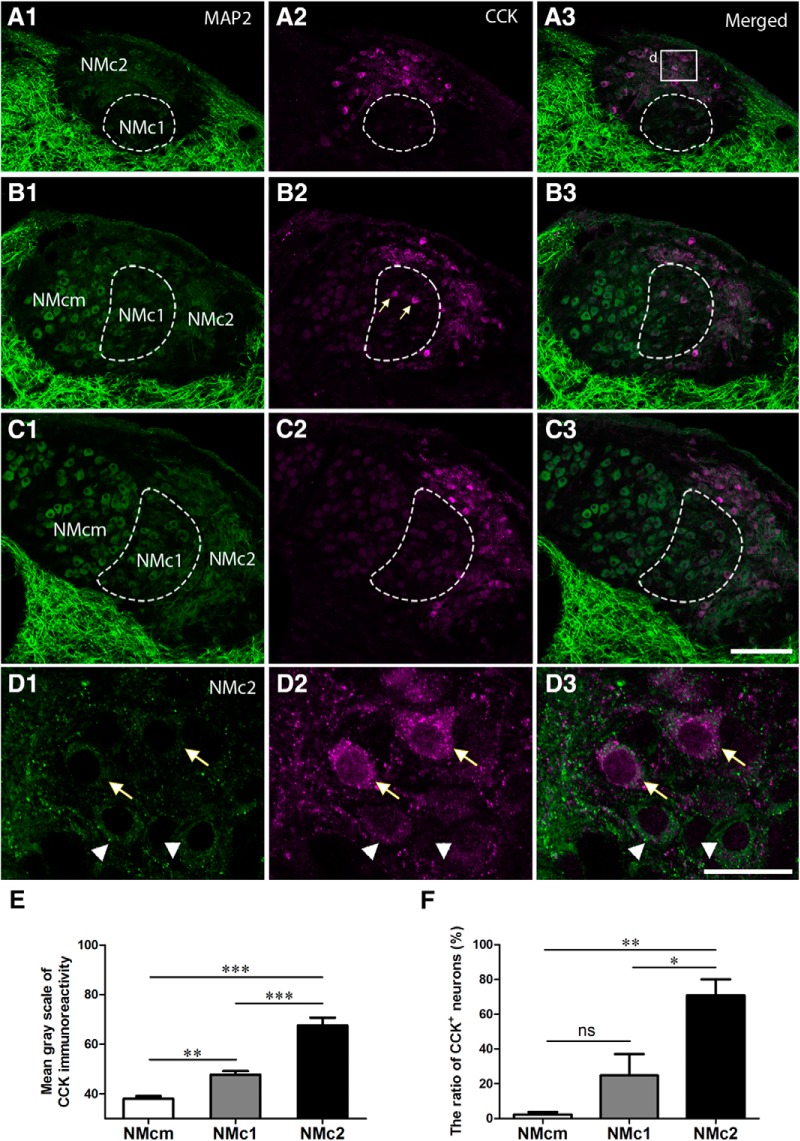
CCK is a biomarker for NMc2. The left (***A1***, ***B1***, ***C1***, ***D1***) and middle (***A2***, ***B2***, ***C2***, ***D2***) columns are MAP2 and CCK immunostaining, respectively. The right column (***A3***, ***B3***, ***C3***, ***D3***) shows the merged images. ***A–C***, Low-magnification images were taken from sections located caudal to rostral from the same animal. Dashed lines outline NMc1. Arrows in ***B2*** indicate CCK-positive neurons in NMc1. ***D***, High-magnification images of the box in ***A3***. Arrows and arrowheads indicate darkly and lightly CCK-labeled NMc2 neurons, respectively. ***E***, Bar chart of the mean grayscale of CCK-expressing neurons in NMcm, NMc1, and NMc2. ***F***, Bar chart of the percentage of CCK-immunoreactive neurons in NMcm, NMc1, and NMc2. *, *P <* 0.05; **, *P <* 0.01; ***, *P <* 0.001; ns, not significant. Data are presented as mean ± SD. Abbreviations: see Fig. 1. Scale bars = 100 μm in ***C3*** (applies to ***A1–C3***) and 20 μm in ***D3*** (applies to ***D1–D3***).

CCK is a broadly expressed peptide hormone in mammalian and avian brains, known as a biomarker for specialized auditory neurons with distinct physiologic properties (Li et al., 2014). Double immunostaining of MAP2 and CCK in the NM demonstrates that CCK immunoreactivity is prominent in NMc2 and was detected in both cell bodies and the most proximate portion of dendrites (Fig. 4). CCK immunoreactivity was also detected in the neuropil regions that are overlapped with MAP2 staining. Although most MAP2-immunoreactive neurons in NMc2 are strongly labeled for CCK (arrows in Fig. 4*D*), some neurons display only background levels of CCK signal (arrowheads in Fig. 4*D*). Occasionally, a few neurons with strong CCK labeling were found in NMc1 (arrows in Fig. 4*B2* ). In contrast, all neurons in NMcm or more rostral portions of NM as well as the majority of the neurons in NMc1 display a low level of staining slightly above the background (Fig. 4*B*, *C*). Statistical analysis confirmed that NMc2 neuronal cell bodies show significantly stronger CCK immunoreactivity (67.5 ± 32.7; *n* = 109 cells from three animals) than those of NMcm (38.0 ± 12.8; *n* = 151 cells from three animals, *p* <0.0001) and NMc1 (47.7 ± 14.4; *n* = 96 cells from three animals, *p* <0.0001; Fig 4*E*). Using 2 SD above the average somatic CCK immunostaining intensity across all measured NMcm neurons as cutoff, the percentage of CCK-positive neurons in NMc2 (70.8 ± 18.4%) is significantly larger than that in NMcm (2.3 ± 2.8%; *n* = 151 cells from three animals, *p* = 0.0012) and NMc1 (24.8 ± 24.5%, *p* = 0.0156; Fig. 4*F*). The difference between NMcm and NMc1 is not significant (*p* = 0.3213), due to the high heterogeneity of NMc1 neurons in CCK expression. These results indicate that NMc2 is distinct from other NM regions in the distribution of CCK.

### NMc1 and NMc2 receive inputs from the auditory nerve

**Figure 5.**
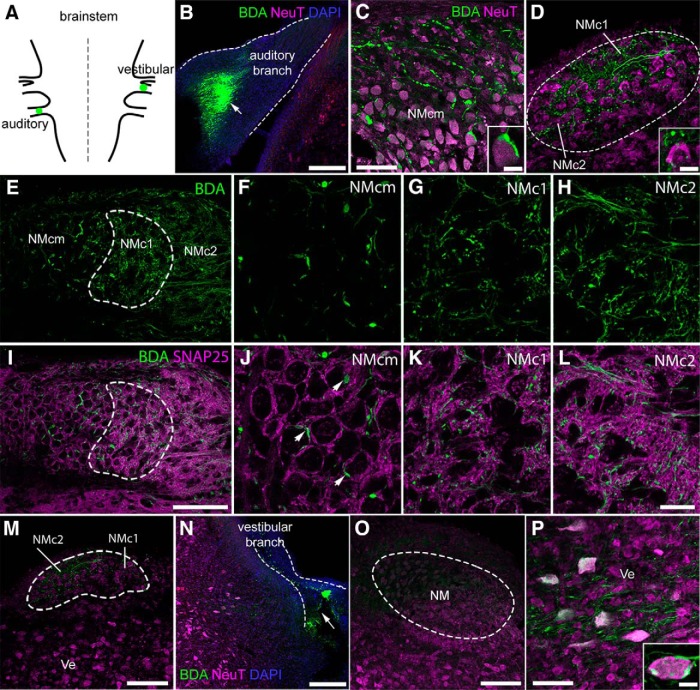
NMc1 and NMc2 receive excitatory inputs from auditory nerve fibers. ***A***, Schematic drawing shows injection sites of BDA in auditory and vestibular branches of the eighth nerve. See details in Materials and Methods. ***B***, The injection site (white arrow) in the cochlear branch outlined by dashed lines. ***C***, BDA-labeled axons and terminals in NMcm. The inset shows the end bulb morphology of a labeled terminal. ***D***, BDA-labeled axons and terminals in NMc1 and NMc2. The images were taken from a section at the level of Fig. 1*E*. The inset shows the bouton-like morphology of labeled terminals. ***E–H***, BDA-labeled axons and terminals in NMcm, NMc1, and NMc2 at the level of Fig. 1*F*. NMc1 is outlined by dashed line. ***F–H*** are closer views of NMcm (***F***), NMc1 (***G***), and NMc2 ***(H***). ***I–L***, Double-labeling of BDA (green) and the excitatory synaptic marker SNAP25 (magenta). ***J–L*** are closer views of NMcm (***J***), NMc1 (***K***), and NMc2 (***L***). Arrows in ***J*** indicate a number of BDA-labeled end bulbs double-stained with SNAP25. ***M***, No labeling was observed in vestibular nuclei after injections in the cochlear branch. ***N***, The injection site (white arrow) in the vestibular branch outlined by dashed lines. ***O***, No labeling in NM after the injection in ***N***. ***P***, BDA-labeled terminals in the vestibular nucleus ventral to NM. Inset shows a labeled terminal around a vestibular neuron. Abbreviations: BDA, dextran; NeuT, NeuroTrace; Ve, vestibular nucleus; NA, nucleus angularis; NL, nucleus laminaris. Other abbreviations: see Fig. 1. Scale bars = 100 μm in ***B***, ***M***, ***N***, ***O***, and P; 50 μm in ***C***, ***D***; 100 µm in ***I*** (applies to ***E*** and ***I***); 20 μm in ***L*** (applies to ***F–H*** and ***J–L***); and 10 μm in insets.

The caudolateral NM was initially considered as a vestibular group in pigeons (Boord and Rasmussen, 1963), and later was reported to be auditory in chickens (Kaiser and Manley, 1996) and barn owls (Köppl and Carr, 1997). To further clarify the nature of NMc1 and NMc2 identified here, we mapped terminal distribution patterns of auditory and vestibular axons of the eighth nerve in brainstem chunk preparations. Because both the auditory and vestibular nerves project exclusively ipsilaterally to the dorsal brainstem (Parks and Rubel, 1978; Kaiser and Manley, 1996), we injected a fluorescent dextran dye in the auditory nerve branch on one side of the brain and made a second injection into the large vestibular nerve branch on the other side of the brain of the same chunk preparations for comparison (Fig. 5*A*).

On the side with injections into the auditory nerve branch (Fig. 5*B*), we found labeled axons and terminals throughout NM including NMc1 and NMc2 (Fig. 5*C–E*). As expected, labeled terminals form large end bulb synapses surrounding the neuronal cell bodies in NMcm and the more rostral portion of NM (Fig. 5*C*, *F*). In contrast, NMc1 and NMc2 contain only bouton-like terminals, which are often found in the space between cell bodies, presumably on dendrites (Fig. 5*D*, *G*, *H*). Double labeling with the synaptosomal-associated protein 25 (SNAP25), a presynaptic marker for excitatory synapses (Oyler et al., 1989; Safieddine and Wenthold, 1999), confirmed that bouton-like terminals in NMc1 and NMc2 are excitatory, similar to end bulbs in NMcm (Fig. 5*I*). As expected, NMcm displays characterized perisomatic staining pattern of SNAP25 (Fig. 5*J*) and intensive neuropil staining in NMc1 and NMc2 (Fig. 5*K*, *L*). No labeled terminals were found in the adjacent vestibular nuclei (Fig. 5*M*). These results demonstrate that NMc1 and NMc2 receive excitatory inputs from the auditory nerves through bouton-like terminals.

On the side with injections into the vestibular branch (Fig. 5*N*), no labeled axons and terminals were found in NM (Fig. 5*O*) and other auditory cell groups in the brainstem including nucleus angularis (NA) and nucleus laminaris (NL). In contrast, we did find labeled terminals in vestibular regions located adjacent to NA and NM (Fig. 5*P*). It is important to note that this observation does not exclude the possibility that NMc1 and NMc2 receive inputs from other vestibular nerve and nuclei.

### NMc1 and NMc2 receive inhibitory inputs from the SON

**Figure 6.**
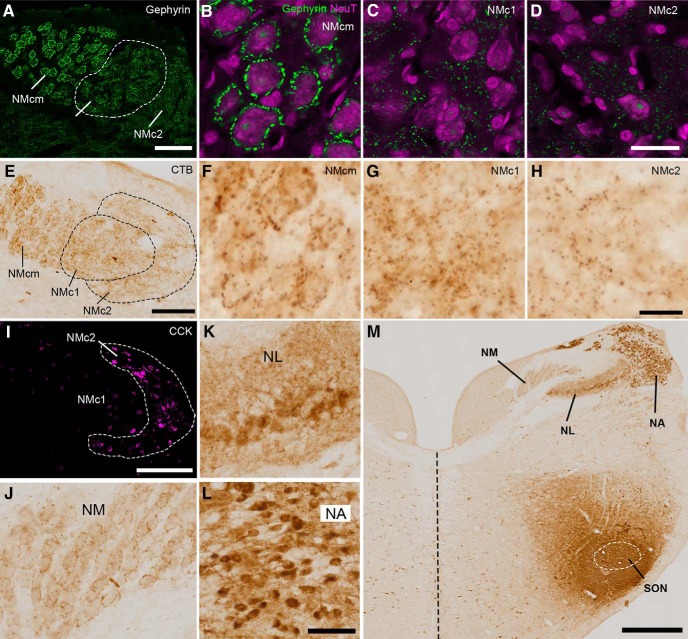
NMc1 and NMc2 receive inhibitory inputs from SON. ***A–D***, Distribution pattern of inhibitory synaptic marker gephyrin. NMc1 is outlined by dashed line. ***B–D*** are high-magnification observations of NMcm (***B***), NMc1 (***C***), and NMc2 (***D***), respectively. ***E–H***, Anterogradely labeled axonal terminals in the caudal NM after *in vivo* injection of CTB into SON. Dashed lines outline NMc1 and NMc2. ***F–H*** are high-magnification observations of NMcm (***F***), NMc1 (***G***), and NMc2 (***H***), respectively. ***I***, Immunostaining of CCK performed on the adjacent section of ***E*** for identifying NMc2. ***J***, Labeled axonal terminals in NM at the level more rostral than NMc. ***K***, ***L***, Labeled cell bodies and neuropil in NL (***K***) and NA (***L***). ***M***, Injection site in SON. White dashed line indicates the approximate border of the SON. The midline is indicated by black dashed line. Abbreviations: CTB, cholera toxin B; SON, superior olivary nucleus. Other abbreviations: see Fig. 1. Scale bars = 100 μm in ***A***, ***E***, ***I***; 50 μm in ***L*** (applies to ***J–L***); 20 μm in ***B*** (applies to ***B–D***) and ***H*** (applies to ***F–H***); and 500 μm in ***M***.

Inhibition is an essential mechanism for precise temporal processing in NM (Burger et al., 2011). To investigate the inhibitory input to the caudal NM, we first examined the distribution pattern of gephyrin, a postsynaptic protein that anchors inhibitory neurotransmitter receptors to the cytoskeleton (Kirsch et al., 1993; Lim et al., 2000). Consistent with previous studies using GABA receptors as an inhibitory synaptic marker (Burger et al., 2005b), gephyrin immunoreactivity forms a perisomatic staining pattern in NMcm (Fig. 6*A*, *B*). This staining pattern is absent in NMc1 and NMc2 (Fig. 6*C*, *D*). Instead, gephyrin immunoreactivity is scattered between the cell bodies in these two regions.

To further identify the source of inhibition in NMc1 and NMc2, we injected CTB, a sensitive neural tract tracer, into the SON *in vivo*. SON receives excitatory input from NA and NL and is the major source of inhibition to NA, NM, and NL (Burger et al., 2005a). Fig. 6 demonstrates a case with CTB injection into a large portion of SON and the surrounding area (Fig. 6*M*). As expected, no labeled cell bodies were detected in NM, whereas labeled terminals were found throughout NM including NMcm, NMc1, and NMc2 (Fig. 6*E*–*H*, *J*). Similar to the staining pattern of gephyrin immunoreactivity, CTB-labeled terminals often surround the cell bodies in NMcm and the higher-frequency region of NM, while displaying a diffused pattern in NMc1 and NMc2. In contrast, both labeled neuropils and cell bodies were found in NA and NL (Fig. 6*K*, *L*).

### NMc1 and NMc2 show differential expression patterns of calcium-binding proteins

Expression of various calcium-binding proteins in auditory neurons displays cell-type specificity and species variation (Takahashi et al., 1987; Rogers, 1989; Li et al., 2013). In chickens, it is reported that all neurons in NM express calretinin but not parvalbumin, two EF-hand calcium binding proteins (Rogers, 1987, 1989). Here we examined the localization of these two proteins in NMc1 and NMc2.

**Figure 7.**
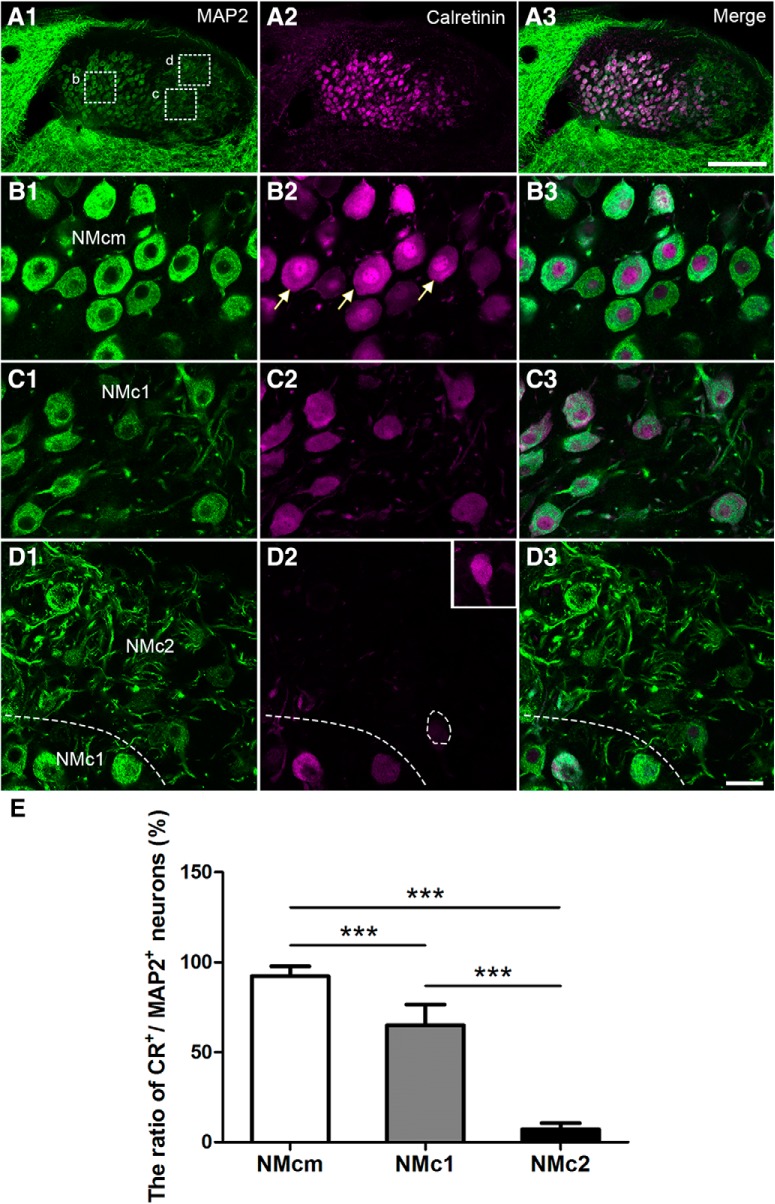
Differential expression of calretinin in the caudal NM. The left (***A1***, ***B1***, ***C1***, ***D1***) and middle (***A2***, ***B2***, ***C2***, ***D2***) columns are MAP2 and calretinin immunostaining, respectively. The right column (***A3***, ***B3***, ***C3***, ***D3***) is the merged images. ***A***, Low-magnification images were taken from a section at the level of Fig. 1*G*. ***B–D***, High-magnification images of the boxes in ***A1***. All images were collected with the same imaging parameters and processed in the same way, except for the inset in ***D2*** in which the brightness is enhanced to show a weakly labeled neuron in NMc2. Note calretinin-expressing neurons in NMcm and NMc1, but not NMc2. The border between NMc1 and NMc2 is indicated by dashed lines in ***D***. ***E***, Bar chart of the ratio of calretinin-expressing neurons in NMcm, NMc1, and NMc2. ***, *P <* 0.001. Data are presented as mean ± SD. Abbreviations: see Fig. 1. Scale bars = 100 μm in ***A3*** (applies to ***A1–A3***) and 20 μm in ***D3*** (applies to ***B1–D3***).

Double labeling of calretinin and MAP2 reveals a highly differential distribution pattern of calretinin in the three NM subregions identified based on the basis of MAP2 staining patterns (Fig. 7*A*). As expected, most neurons in NMcm (∼91%) and the more rostral portion of NM display strong somatic staining of calretinin in the cytoplasm, although the staining intensity varies across neurons (Fig. 7*B*, *E*). In many neurons, significant staining in the nucleus is also present and often more intense than the cytoplasmic staining (arrows in Fig. 7*B2*). Approximately 66% of neuronal cell bodies in NMc1 are calretinin immunoreactive, although the staining intensity is generally lower than that of neurons in the adjacent NMcm (Fig. 7*C*, *E*). In NMc1, calretinin staining intensities in the nucleus and cytoplasm are largely comparable. Calretinin-labeled dendrites are also seen in this region. In contrast, calretinin immunoreactivity is strikingly low in NMc2 (Fig. 7*D2* ). Only 8% of neuronal cell bodies in NMc2 display weak calretinin immunostaining above the background level, and these neurons are often located close to NMc1 (dashed lines and inset in Fig. 7*D2*, *E* ). A sharp border between NMc1 and NMc2 is clear based on calretinin immunostaining. Statistical analysis further verified the differential expression pattern of calretinin along the tonotopic axis. The percentage of calretinin-positive neurons in NMcm (91 ± 5%; *n* = 426 cells from three animals) is significantly larger than that in NMc1 (66 ± 11%; *n* = 397 cells from three animals, *p* < 0.001) and NMc2 (8 ± 3%; *n* = 474 cells from three animals, *p* < 0.001). In addition, the percentage of calretinin-positive neurons in NMc1 is also significantly larger than that in NMc2 (*p* < 0.001; Fig. 7*E*).

**Figure 8.**
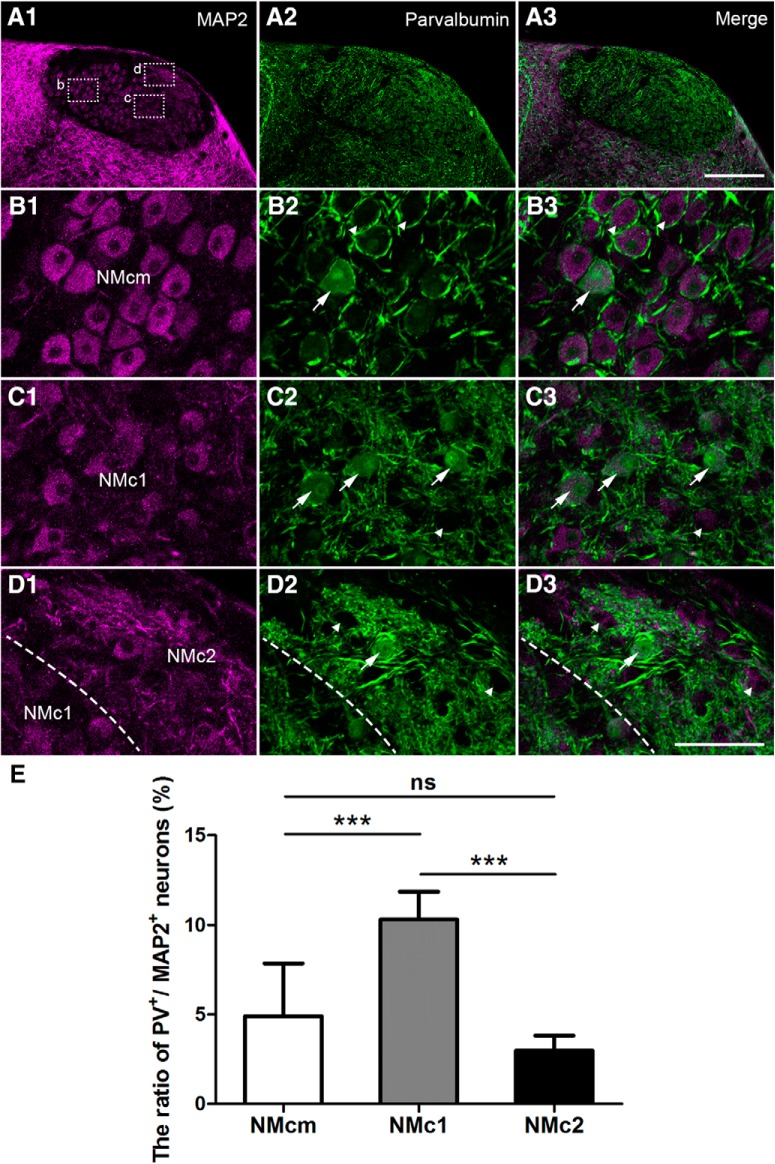
Differential expression of parvalbumin in the caudal NM. The left (***A1***, ***B1***, ***C1***, ***D1***) and middle (***A2***, ***B2***, ***C2***, ***D2***) columns are MAP2 and parvalbumin immunostaining, respectively. The right column (***A3***, ***B3***, ***C3***, ***D3***) is the merged images. ***A***, Low-magnification images were taken from a section at the level of Fig. 1*G*. ***B–D***, High-magnification images of the boxes in ***A1***. All images were collected with the same imaging parameters and processed in the same way. Arrows and arrowheads in ***B–D*** indicate labeled and unlabeled somata for parvalbumin. The border between NMc1 and NMc2 is indicated by dashed lines in ***D***. ***E***, Bar chart of the ratio of parvalbumin-expressing neurons in NMcm, NMc1, and NMc2. ***, *P <* 0.001; ns, not significant. Data are presented as mean ± SD. Abbreviations: see Fig. 1. Scale bars = 100 μm in ***A3*** (applies to ***A1–A3***) and 50 μm in ***D3*** (applies to ***B1–D3***).

Double-labeling of MAP2 and parvalbumin (Fig. 8) provided a strikingly different pattern. Low-magnification images show strong parvalbumin immunoreactivity throughout NM (Fig. 8*A*). Closer views of NMcm and the more rostral NM reveal intense neuropil staining surrounding unstained NM cell bodies (arrowheads in Fig. 8*B*). These parvalbumin-labeled processes resemble the end bulbs of the auditory nerve in morphology and location. Neuropil staining is also abundant in NMc1 and NMc2, primarily present as neuronal processes of small caliber. A small population of MAP2-labeled cell bodies were double-labeled for parvalbumin (arrows in Fig. 8*B*–*E*). They were encountered more frequently in NMc1 than in NMcm and NMc2. Statistical analysis on population data across sections and animals further confirmed that the percentage of parvalbumin-positive neurons in NMc1 (10.3 ± 1.6%; *n* = 275 cells from three animals) is significantly larger than that in NMcm (4.9 ± 3.0%; *n* = 473 cells from three animals, *p* < 0.001) and NMc2 (3.0 ± 0.9%; *n* = 297 cells from three animals, *p* < 0.001). In addition, the percentage of parvalbumin-positive neurons in NMcm is comparable to that in NMc2 (*p* = 0.569; Fig. 7*E*). Together, NMc1 contains neurons expressing calretinin and parvalbumin, whereas most neurons in NMc2 do not express these two proteins at a detectable level.

### NMc1 and NMc2 neurons show distinct passive and active membrane properties

**Figure 9.**
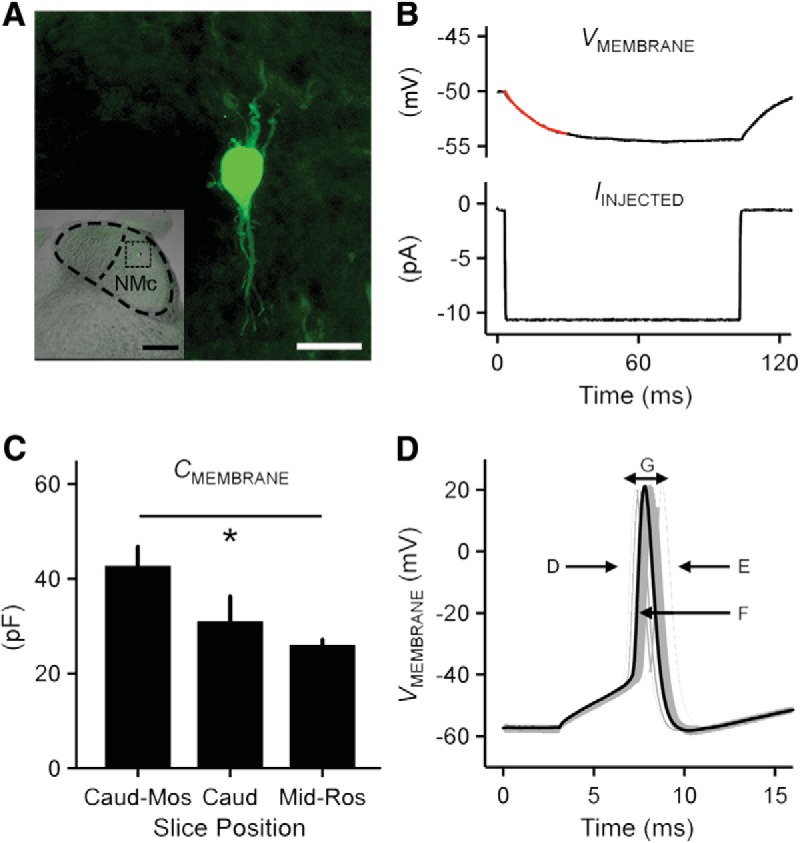
Electrophysiological protocols applied to NMc1 and NMc2 neurons. ***A***, Neurobiotin-labeled NMc1/NMc2 neuron. Inset shows low-magnification image of the entire coronal NM region with the labeled NMc1/NMc2 neuron. Dorsal, top; lateral, right. Scale bar = 20 μm (200 μm in inset). ***B***, Current clamp protocol to measure passive membrane properties. Upper trace shows the representative voltage response (average of 30 repetitive trials) recorded from an NMc1/NMc2 neuron in response to a hyperpolarizing current injection (lower trace, –10 pA). A single exponential was fitted to a 30-ms time window after the current injection (superimposed red line), to calculate time constant (tau), input resistance, and membrane capacitance. ***C***, Population data showing membrane capacitance (*C*_MEMBRANE_) sampled from the first (also referred as caudomost [caud-mos]) slice, second/third slices (caud), and middle to rostral slices (mid-ros, mid- to high-frequency NM; data modified from Hong et al. [2016]). Asterisk represents significance at *p* < 0.05. Error bars show SE. ***D***, Metrics used to measure AP properties. Representative first APs (30 superimposed trials) were recorded from an NMc1/NMc2 neuron in response to current injections with the strength 25% above the threshold current (duration 100 ms). Several AP properties were characterized: rise rate (***D***), fall rate (***E***), half width (***F***), and reliability range (***G***). Population data of AP properties are shown in the corresponding panels in Fig. 11.

We explored passive and active membrane properties of NMc1/NMc2 neurons, and when appropriate, compared them with mid- to high-frequency NM neurons. Neuronal location and morphology were confirmed using neurobiotin for a subset of experiments. An example of a neurobiotin-labeled neuron is shown in Fig. 9*A*. This neuron was located within the region of NMc1 and NMc2; lateral to NMcm (inset) and contained multiple dendritic processes. Despite the clear anatomic distinctions noted above, from an electrophysiological perspective we were not able to differentiate NMc1 and NMc2 neurons. Instead, we used membrane capacitance as an index for neuron size (i.e., surface area) with the idea that NMc2 neurons would present with a larger membrane capacitance than NMc1 neurons. An example of the recording protocol and membrane response is shown in Fig. 9*B* (see Materials and Methods for calculation of membrane capacitance). We found the following evidence that supports the use of membrane capacitance as an indicator of neuronal size. First, individual brainstem slices were placed in a custom chamber that maintained the tonotopic gradient, from the caudomost slice to the rostromost slice representing slices 1–7, respectively. As mentioned in Materials and Methods, the caudomost two to three slices were used for NMc recordings. According to our anatomic data (see Fig. 1*A*, *E*), NMcm neurons are not observed in the caudomost slice. The majority of neurons in the caudomost slice are NMc2 neurons, and indeed are present with a larger estimated membrane capacitance (Fig. 9*C*, caud-mos). Also, when compared with mid- to high-frequency NM neurons (e.g., neurons taken from slices shown in Fig. 1*C*, *D*, Fig. 9*C*, mid-ros; Hong et al., 2016), NMc2 neurons have significantly larger membrane capacitance (42.83 ± 10.50 vs. 26.15 ± 4.60 pF). The difference in membrane capacitance is likely due to extensive dendrites (despite smaller soma) of NMc2 neurons compared with adendritic NM neuron (despite larger soma).

Second, when NMcm neurons begin to gradually appear, the second and third slice contains both NMc1 and NMc2 neurons (see Fig. 1*B*, *F*, *G*). *Post hoc* testing of membrane capacitance did not result in significant difference between neurons taken from the second and third caudal slices (Fig. 9*C*, caud slices) compared with neurons taken from the caudomost slice, albeit NMc2 neurons presented with a larger membrane capacitance on average. The lack of significance is likely due to the intermingled distribution pattern of NMc1 and NMc2 in the caudal slices. Additionally, membrane capacitance of second and third caudal slices is not significantly different from that of higher-frequency NM (Fig. 9*C*, mid-rostral slices), likely because membrane capacitance of mid- to high-frequency NM neurons is relatively homogeneous, i.e., the capacitance values for individual neurons vary minimally from the average (26.15 ± 4.6 pF, Hong et al., 2016). This result is in line with homogeneity of NM neuronal size. In contrast, we observed a large variability for NMc neurons. In particular, five NMc neurons obtained from the second caudal slice presented with smaller capacitance than the average of mid- to high-frequency NM neurons. Their smaller membrane capacitance is reminiscent of properties of NMc1 neurons located adjacent to the NMcm region, which show significantly smaller somatic area and minor dendritic processes (see Figs. 2 and 3*C*, *G* ). As a result, these NMc1 neurons showed even smaller capacitance than traditional NM neurons and thus led to the nonsignificance reported in Fig. 9*C*. Taken together, membrane capacitance is a relatively reliable measurement of neuronal size for NMc neurons. NMc1 neurons present with smaller membrane capacitance. This is likely due to their smaller soma and less complex dendritic arborization (compared with NMc2: see Fig. 3), whereas NMc2 neurons usually present with larger membrane capacitance owing to their more extensive dendritic processes.

We compared membrane capacitance among NMc1/NMc2 neurons and examined whether neurons with larger membrane capacitance (i.e., likely NMc2 neurons) show distinct intrinsic properties compared with neurons with smaller membrane capacitance (i.e., likely NMc1 neurons), serving as an indirect method to classify NMc1 and NMc2 AP properties. It should be noted that comparisons of all passive membrane properties (i.e., resting membrane potential, time constant, input resistance, and membrane capacitance) were made under the same experimental conditions (e.g., room temperature) and were significantly different from higher-frequency NM neurons (Table 2).

**Table 2.** Comparison of passive membrane and AP properties between NMc1/NMc2 and mid- to high-CF NM neurons

Property	NMc1/NMc2 (*n*)	Mid- to high-CF NM*^a^*	*P*, *t* test
Passive membrane properties			
RMP (mV)*^b^*	–50.55 ± 9.74 (30)	–66.52 ± 8.49 (28)	*<*0.0001
Time constant tau (ms)	20.39 ± 17.25 (29)	3.18 ± 1.33 (20)	*<*0.0001
Input resistance (MΩ)	467.2 ± 342.5 (29)	123.90 ± 49.90 (20)	*<*0.0001
Membrane capacitance (pF)	41.25 ± 21.74 (29)	26.15 ± 4.60 (20)	*<*0.01
Action potential properties			
Threshold current (pA)	38.96 ± 25.96 (22)	321.70 ± 121.00 (28)	*<*0.0001
Max rise rate (mV/ms)*^c^*	136.1 ± 41.01 (22)	155.60 ± 42.19 (28)	0.107
Max fall rate (mV/ms)*^c^*	–69.14 ± 22.16 (22)	–104.40 ± 29.79 (28)	*<*0.0001
AP half width (ms)*^c^*	1.45 ± 0.48 (22)	0.97 ± 0.17 (28)	*<*0.0001
AP reliability range (ms)*^c^*	6.98 ± 5.87 (17)*^d^*	0.21 ± 0.14 (28)	*<*0.0001

a Data from Hong et al., 2016. Experimental conditions (e.g., temperature) and recording parameters (e.g., membrane capacitance) for both studies are the same.

b Numeric values without the correction of –10 mV junction potential.

c Measured from APs in response to current injections 25% above threshold current.

d Five outliner neurons with reliability range >30 ms were removed.

AP properties of interest are highlighted in Fig. 9*D* from a representative E21 neuron. To compare AP properties across different NM regions, APs were evoked using a sustained current injection (100 ms) set at 25% above threshold current. Three variables were analyzed regarding AP kinetics: maximal rise rate, fall rate, and half width. Rise and fall rates were calculated as the maximum rate of increase and decay in the AP-depolarizing and repolarizing phase, respectively. Half width was quantified as AP duration measured at half of the maximum amplitude relative to the resting membrane potential. To quantify AP reliability, we stimulated neurons using sustained suprathreshold current depolarization (i.e., 25% above threshold current) across 30 trials (interpulse stimulus intervals, 2 s) and calculated the range of time points of AP peak occurrence.

### NMc1 and NMc2 neurons show distinct and heterogeneous AP properties

A biophysical hallmark of mid- to high-frequency NM neurons is the generation of a single-onset AP in response to sustained depolarization (Reyes et al., 1994; Howard et al., 2007; Hong et al., 2016). We found several AP properties of NMc1 and NMc2 neurons that were notably different from this biophysical phenotype of NM neurons. These include increased excitability and slower, less reliable APs (Table 2). In the following sections, we report these differences in greater detail.

**Figure 10.**
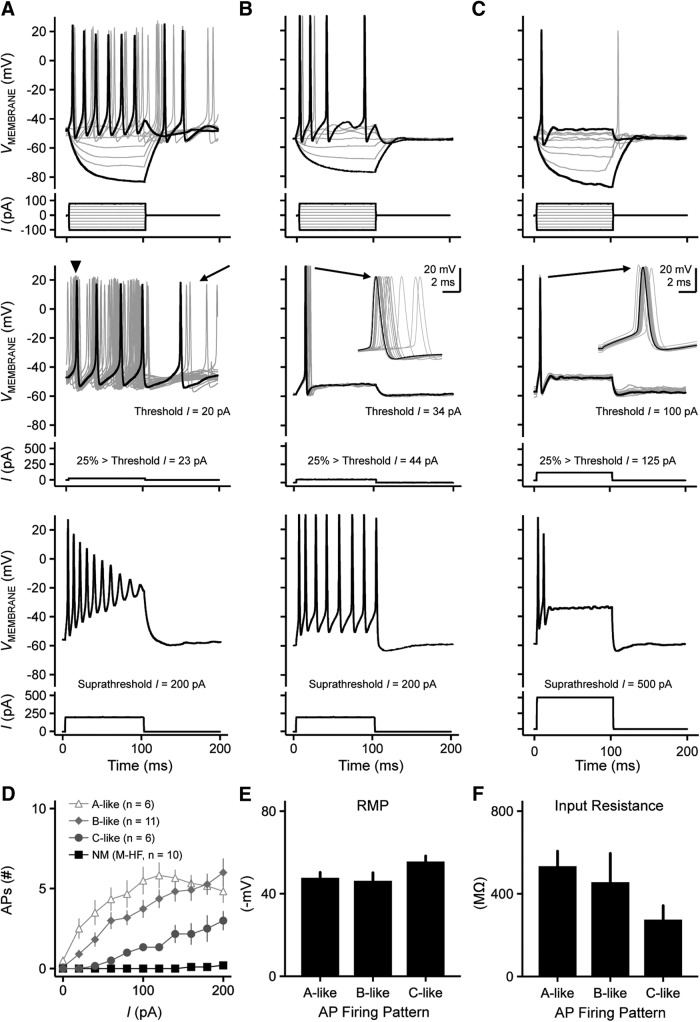
Heterogeneous voltage responses to current injections recorded from NMc1 and NMc2 neurons. ***A***, ***B***, ***C***, (top) Representative voltage responses recorded from three NMc1/NMc2 neurons to current injections from –100 to 80 pA in steps of 20 pA (bottom). (middle) Representative voltage responses (30 superimposed trials) recorded from the same three NMc1/NMc2 neurons shown in the top panel, respectively. Bottom, current injections with the strength 25% above threshold current. Arrowhead in ***A*** shows widespread AP peak occurrences for this neuron. Arrow in ***A*** shows spontaneous activity. Insets in ***B*** and ***C*** show the enlargement of 30 superimposed APs. (bottom) Representative voltage responses recorded from three NMc1/NMc2 neurons to current injections with the strength of 200 pA (***A*** and ***B***) or 500 pA (***C***). ***D***, Population data showing the number of APs elicited as a function of current injections from 0 to 200 pA, steps of 20 pA. NMc1/NMc2 neurons are divided into three subgroups: neurons displaying voltage responses similar to the neurons shown in ***A***, ***B***, and ***C*** are noted as A-like, B-like, and C-like, respectively (see Results for objective classification details). Mid- to high-frequency NM neurons (M-HF) are also shown as a reference (data modified from Hong et al., 2016). ***E***, ***F***, Population data showing resting membrane potential (RMP, ***E***) and input resistance (***F***) of A-like, B-like, and C-like NMc1/NMc2 neurons. The duration of all current injections in this figure is 100 ms. Error bars show SE.

Fig. 10*A–C* shows representative recordings from three different NMc1/NMc2 neurons. Using current commands ranging from –100 pA to 80 pA, NMc1/NMc2 neurons fired a range of APs to sustained suprathreshold depolarization, from multiple spikes to a single spike (Fig. 10 *A–C*, top, respectively). Threshold current required to elicit an AP was approximately an order of magnitude lower for NMc1/NMc2 compared with higher-frequency NM (Table 2). For all three NMc1/NMc2 neurons shown in Fig. 10, the threshold current was <110 pA, with the lowest current being 20 pA (Fig. 10*A*). NMc1/NMc2 neurons presented with a range of excitability when a suprathreshold current command 25% above threshold current was applied. For the neuron shown in Fig. 10*A*, a weak depolarizing current injection of 23 pA resulted in sustained AP firing with highly variable first spike occurrence and considerable spontaneous activity long after the completion of the sustained current (Fig. 10*A*, middle, arrowhead and arrow, respectively). With a suprathreshold current injection of 200 pA, the neuron responded with multiple spikes that continuously declined in AP amplitude during the duration of the stimuli (Fig. 10*A*, bottom). The AP amplitudes halfway through the sustained current injection (i.e., 50 ms after the stimulus onset) were reduced by 36% on average (*n* = 6), and these neurons presented with depolarization block at the end of the injected current time window.

These results were partially true for the neuron shown in Fig. 10*B*. When sustained current injections ranging from –100 pA to 80 pA were applied, strengths >60 pA resulted in multiple spiking (Fig. 10*B*, top) but a current injection 25% above threshold (i.e., 44 pA) resulted in a single-onset AP (Fig. 10*B*, middle). The time of the “single-spike” peak occurrence across 30 trials was highly variable (i.e., large AP reliability range, inset). For the population of neurons that presented with this response property (*n* = 11), AP reliability range was significantly larger compared with mid- to high-frequency NM neurons (5.28 ± 4.41 ms, *p* < 0.0001; Table 2). In addition, this neuron fired tonically throughout the duration of the suprathreshold current injection (i.e., 200 pA) with no rundown of AP amplitudes (Fig. 10*B*, bottom).

The single AP phenotype that is typical of mid- to high-frequency NM was also occasionally observed. For the neuron shown in Fig. 10*C*, current injections ranging from –100 to 125 pA resulted in a single AP (top and middle, respectively), but the time of peak AP occurrence was somewhat variable (inset) and not as reliable compared with higher-frequency NM neurons (4.48 ± 4.72 ms, *p* < 0.0001; Table 2). Interestingly, when we systematically increased the strength of current injections beyond the 25% criteria, all of these single-spiking neurons generated multiple APs (Fig. 10*C*, bottom; Fig. 10*D*). Only one-quarter of recorded neurons (6 of 23) resulted in this response property, suggesting that a subpopulation of NMc1/NMc2 neurons resembles some aspects of mid- to high-frequency NM neurons, albeit minimally.

Regardless of the heterogeneity of active membrane properties, all NMc1/NMc2 neurons increased their AP output as a function of the increasing strength of current injection, which is markedly distinct from traditional NM (Fig. 10*D*). As such, we used the neuron’s input/output function to objectively categorize a neuron’s firing pattern into three types. The A-like neurons (in reference to Fig. 10*A*) and the B-like neurons (in reference to Fig. 10*B*) both generated multiple APs at the moderate current levels of ∼80 pA. With increasing current strength, the A-like neurons responded in a nonmonotonic fashion that resulted in reduced spike output at higher current strengths (e.g., 200 pA; Fig. 10*D*). In contrast, the B-like neurons fired multiple APs (>6) in a monotonic fashion with increasing current strength (Fig. 10*D*). Finally, the C-like neurons (in reference to Fig. 10*C*) generated a single AP to moderate current injections but fired several APs (<4) in a monotonic fashion to increasing current strength (Fig. 10*D*). It should be noted that the firing of multiple APs during sustained depolarization is not observed in late-developing neurons of surrounding temporal coding brainstem nuclei (e.g., NL and more rostromedial NM), regardless of the strength and duration of current injection (Fig. 10*D*; Hong et al., 2016).

It should also be noted that the different spiking activity was not due to differences in neuronal integrity of NMc1/NMc2 neurons. Across the three NMc groups, we found no significant differences in resting membrane potential (RMP; Fig. 10*E*, *p* = 0.21) and input resistance (Fig. 10*F*, *p* = 0.25), both of which are indicators of neuronal integrity. Despite the relative homogeneity of NMc neuronal integrity, C-like neurons did show a more hyperpolarized RMP and lower input resistance, suggesting that their underlying ion channel conductances might differ from other NMc neurons.

**Figure 11.**
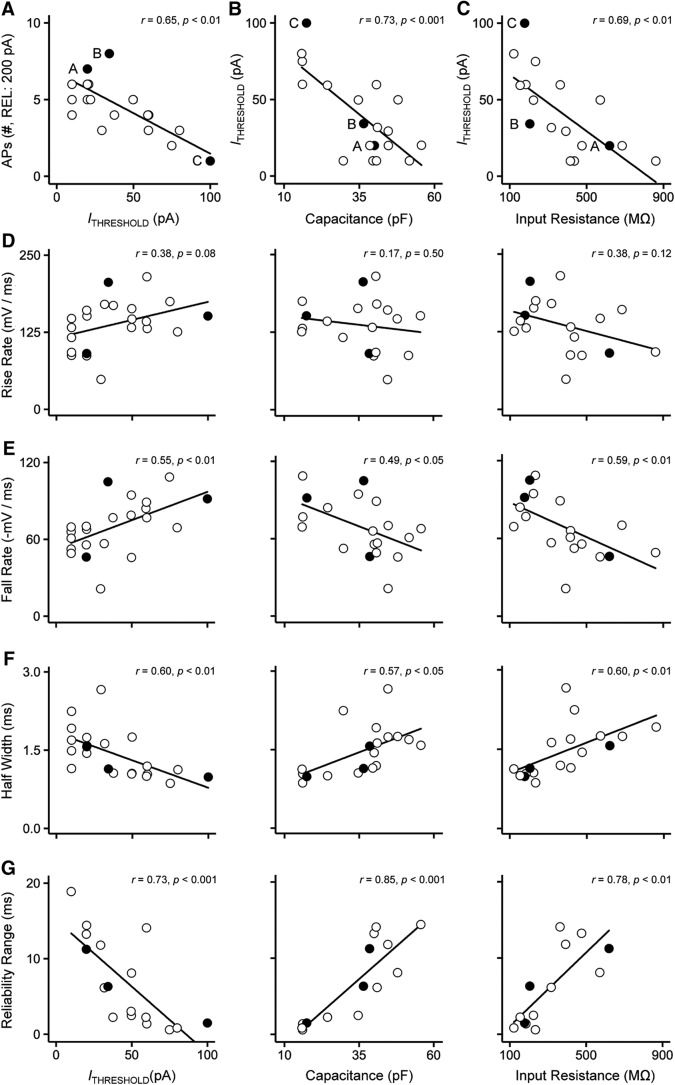
Heterogeneity of AP properties of NMc1 and NMc2 neurons. ***A***, Number of APs generated in response to 200-pA current injections plotted as a function of threshold current for individual NMc1/NMc2 neurons. Three filled and labeled circles represent the neurons shown in Fig. 10*A, B*, and *C*, respectively. Correlation coefficient *r* and *p* values are shown. ***B***, ***C***, Threshold current plotted as a function of membrane capacitance (***B***) and input resistance (***C***) for individual NMc1/NMc2 neurons. ***D–G***, Population data of AP rise rate (***D***), fall rate (***E***), half width (***F***), and reliability range (***G***) are plotted for individual NMc1/NMc2 neurons, as a function of threshold current (left), membrane capacitance (middle), and input resistance (right). Correlation coefficient *r* and *p* values are shown. Three filled circles represent the neurons shown in Fig. 10*A, B*, and *C*, respectively. Note that in ***G***, five outliers with extremely large range (>30 ms) were removed.

Based on these observations, we speculate that there is a population gradient of active membrane properties that results in diverse firing patterns among NMc1/NMc2 neurons. This speculation is supported by the significant correlation between the number of APs generated and threshold current (Fig. 11*A*), along with the significant correlation between threshold current and input resistance (Fig. 11*C*). These results indicate that NMc1/NMc2 neurons with lower threshold currents can fire multiple APs to sustained depolarization (i.e., more excitable than those with higher threshold currents) and have a higher input resistance. This is consistent with the expression gradient of K_V_1.1, the alpha subunit associated with the low-voltage activated potassium channel responsible for single-spiking behavior (Fukui and Ohmori, 2004). Because the low-voltage activated potassium channels generate an outward current partially activated at rest (Rathouz and Trussell 1998; Howard and Rubel 2010), one would expect that less excitable neurons also exhibit lower input resistance than those with higher excitability. Indeed, the C-like NMc neurons have a lower input resistance and more hyperpolarized RMP than the A-like and B-like neurons (Fig. 10*E*, *F*). This is further supported by the significant correlation between threshold current and membrane capacitance (Fig. 11*B*), indicating that NMc2 neurons have larger surface area and are more excitable than NMc1. Not surprisingly, we found a significant correlation between membrane capacitance and input resistance (*r* = 0.66, *p* < 0.01, data not shown). Therefore, we used threshold current, membrane capacitance, and input resistance as indices of excitability for individual NMc1/NMc2 neurons and explored whether this gradient of excitability affects AP properties using correlation analyses.

We did not find a significant correlation with AP rise rate for threshold current, membrane capacitance, or input resistance (Fig. 11*D*), indicating that rise rate is less prone to population gradient of excitability. This is further supported by the nonsignificant difference in rise rate between NMc1/NMc2 and higher-frequency NM neurons (Table 2). In contrast, AP fall rate was significantly correlated with threshold current, membrane capacitance, and input resistance (Fig. 11*E*). Neurons with larger capacitance, lower thresholds, and higher input resistance (i.e., NMc2 neurons) are more likely to have slower repolarization. As a result, AP half width was also significantly correlated with all three variables (Fig. 11*F*), indicating that larger, more excitable and less permeable neurons (i.e., NMc2 neurons) have wider APs. Finally, AP reliability range was also significantly correlated with threshold current, membrane capacitance, and input resistance (Fig. 11*G*), indicating that leakier neurons with less surface area and higher threshold currents (i.e., NMc1 neurons) generate more temporally reliable APs.

To summarize, we observed clear heterogeneity of evoked activity for NMc1/NMc2 neurons. Ongoing experiments are determining the underlying synaptic and intrinsic mechanisms and whether this heterogeneity is associated with anatomically and biochemically distinct neuronal types.

## Discussion

**Figure 12.**
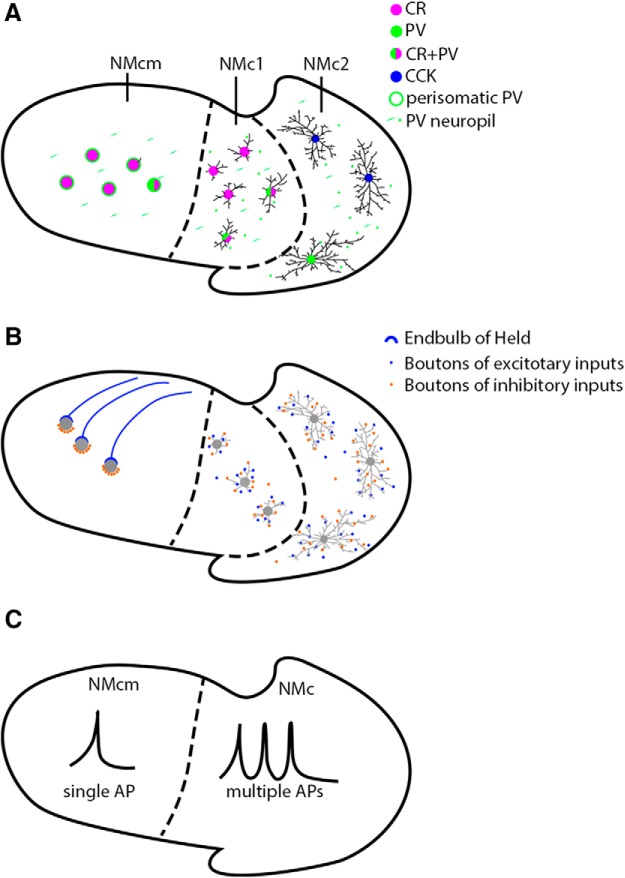
Summary drawings of neuronal features in caudal NM. Based on cytoarchitecture, the caudal NM (regions outlined by solid black lines) is divided into three subdivisions, NMcm, NMc1, and NMc2. Borders between subregions are indicated by dashed lines. Left is medial and up is dorsal. ***A***, Morphology and molecular signatures. NMcm, NMc1, and NMc2 neurons exhibit different dendritic complexity and cell body size. Compared with NMcm neurons with few dendrites, NMc1 and NMc2 neurons preserve more dendrites. Notably, NMc2 neurons show longer total dendritic branch lengths than NMc1. On average, NMcm are larger than NMc1 and NMc2 neurons in somatic size, and the majority of cells with the smallest cell body sizes are located in NMc2. Moreover, neurons in the three subregions also show distinct expression patterns of calretinin (magenta), parvalbumin (green), and CCK (blue). Most neurons in NMcm and NMc1 express calretinin, whereas neurons in NMc2 are not immunoreactive for this protein. A substantial number of NMc1 neurons coexpress calretinin and parvalbumin (half green and half magenta), but only a few neurons in NMcm and NMc2 show parvalbumin expression. Whether CCK-positive neurons in NMc2 are immunoreactive for parvalbumin or calretinin is not determined. Blue circles only represent CCK immunoreactivity of NMc2 neurons, not indicating restricted expression in cell bodies. Extensive neuropil staining of parvalbumin (short green lines) is observed in all three subregions. In NMcm, parvalbumin neuropil staining shows perisomatic pattern (green rings), whereas in NMc1 and NMc2, parvalbumin-positive neuropils (green spots) scatter between cell bodies. ***B***, Connectivity. Neurons in NMcm receive excitatory (blue) and inhibitory (orange) inputs via end bulbs and small boutons, respectively. The inhibitory inputs form bouton synapses on the cell bodies. In contrast, NMc1 and NMc2 neurons receive both excitatory and inhibitory inputs via bouton terminals, primarily in the neuropil (presumably dendrites). ***C***, Biophysics. NMcm neurons generate single-onset AP in response to sustained depolarization, whereas NMc1/NMc2 neurons display the ability of generating multiple action potentials to suprathreshold sustained depolarization and are spontaneously active. Abbreviations: see Fig. 1 for anatomic terms. CR, calretinin; PV, parvalbumin; CCK, cholecystokinin.

The current study characterizes multiple neuron types in the caudolateral region of the avian cochlear nucleus, nucleus magnocellularis (NMc1 and NMc2). These neuron types display unique cellular morphology, molecular signatures, and biophysics, distinct from traditional definitions of NM neurons (Fig. 12). Below we compare the structural and functional properties of NMc1 and NMc2 neurons with previous studies in the caudolateral NM and discuss potential mechanisms underlying these unique properties.

### Definition of NMc

The chicken NMc, as identified in this study, is the most caudolateral portion of NM, where neurons possess extensive dendrites and synapses with small bouton-like axonal terminals from the auditory nerve. In contrast, well-characterized neurons located in the major body of NM, which is rostromedial to NMc, lack substantial dendritic structure and are innervated by large somatic synapses (i.e., the end bulbs of Held) from the auditory nerve.

NMc represents the low-frequency range of the avian tonotopic axis. According to the tonotopic organization of the chicken NM (Rubel and Parks, 1975), NM neurons with characteristic frequency (CF) from 170 to 4100 Hz are located progressively from caudolateral to rostromedial. Because of the technical limitation of that study, which did not generate acoustic stimuli below 100 Hz, CF ranges in a most caudolateral region were not determined, giving rise to the initial notion that this NM region is nonauditory. Subsequent studies did record NM neurons approximately in the caudal region that respond to tones as low as 10 Hz in frequency, although their exact location was not specifically mapped in relationship to the tonotopic organization (Warchol and Dallos, 1990). Consistently, behavioral studies have confirmed that chickens hear as low as 2 Hz (Hill et al., 2014). Combined tract tracing and physiologic studies further demonstrated that the NM region containing bouton terminals corresponds to frequencies <500 Hz of the tonotopic map (Fukui and Ohmori, 2004). Taking these findings together, we propose that NMc in chickens contains CFs <500 Hz and is divided into NMc1 and NMc2. NMc1 corresponds to the low-frequency NM defined by Fukui and Ohmori (2004) with approximate CFs of 100–500 Hz, and NMc2 region coincides with even lower CFs <100 Hz that was previously considered nonauditory in chickens by Rubel and Parks (1975) as well as in pigeons (Boord and Rasmussen, 1963). Further *in vivo* recordings are needed to map the precision of the tonotopic organization in these two regions and the exact location of NM neurons with CFs <100 Hz.

An important question is whether there is a clear boundary between NMc1, NMc2, and the remaining NM. NM is known to have gradients in cellular morphology and physiology along the tonotopic axis (Rubel and Fritzsch, 2002). Indeed, when looking at single metric (dendritic arborization, synaptic terminal morphology, or physiologic response), the caudal NM gradually increases the magnitude of its divergence from the relatively homogeneous morphology and physiology observed in more rostral NM. We suggest, however, that the distinct morphologic and physiologic features of the regions we have called NMc1 and NMc2 produce break points in the continuum to form distinct groups of cells that could possibly have distinct functions or adopt distinct mechanisms for accomplishing similar functions. Although the borders are blurry with a number of single metric, the break points are clear when considering the composite anatomic and physiologic profiles of the neurons, particularly when combined with the fairly discrete variations in protein expression. This proposal is also supported by studies in barn owls, another avian species in which the low-frequency NM has been studied (Takahashi and Konishi, 1988; Köppl, 1994; Köppl and Carr, 1997). The barn owl low-frequency NM, similarly defined as a caudolateral region containing long dendrites and bouton-like synapses, displays two unique cell types with distinct dendritic morphology, consistent with the heterogeneity of the chicken NMc.

### Differential expression of calcium-binding proteins in NM

In addition to structural specializations, our data further reveal that NMc1 and NMc2 neurons express a unique set of calcium-binding proteins and neuropeptides. Previous studies reported that the chicken NM neurons express calretinin but not parvalbumin (Rogers, 1989; Parks et al., 1997; Stack and Code, 2000). Our data confirmed this expression pattern in the adendritic portion of NM, but we found that most neurons in NMc2 do not express calretinin. This difference is likely due to possible overlook of this caudal cell group with negative calretinin staining in previous studies. Lack of calretinin expression in NMc2 is consistent with calretinin-negative neurons in the ventral NA (low frequency; Bloom et al., 2014), indicating this maybe a common feature for the low-frequency neurons in the chicken cochlear nuclei. In addition, we found parvalbumin-expressing neurons in NM including NMc1 and NMc2. Similarly, the majority, if not all, of NM neurons coexpress these two calcium-binding proteins in the zebra finch (Li et al., 2013), emu (MacLeod et al., 2006), and owl (Takahashi et al., 1987; Kubke et al., 1999). Calretinin and parvalbumin have fast and slow calcium-binding kinetics, respectively, which are fine-tuned by instantaneous intracellular calcium concentration. They can work together to modulate global and local intracellular calcium signals in the same cell (Dargan et al., 2004). Differential expression of various calcium-binding proteins has been associated with cell type–specific calcium regulation and cellular physiology (Nejatbakhsh and Feng, 2011). In this study, we observed that the percentage of calretinin or parvalbumin expressing neurons are comparable between NMc1 and NMcm, suggesting that the chicken NMc1 may share some common mechanisms with NM neurons in calcium regulation. On the other hand, these calcium-binding proteins may not be critical for NMc2 neurons.

It is important to point out that calretinin plays an important role in modulating neuronal excitability. In calretinin-knockout mice, GABAergic interneurons in the hippocampus express excess GABA, which leads to impaired long-term potentiation induction of dentate gyrus cells (Schurmans et al., 1997). Similarly, cerebellar granule cells lacking calretinin show increased excitability, indicated by faster action potentials and repetitive spike discharges (Schiffmann et al., 1999; Gall et al., 2003; Bearzatto et al., 2006). Importantly, these calretinin-deficient induced changes are rescued by administering BAPTA, a buffer with fast calcium-binding capability, further suggesting that calretinin reduces neuronal excitability via fast calcium binding (Gall et al., 2003). Many neurons in the chicken NMc do not express calretinin and are more excitable, suggesting that the absence of calretinin may contribute to this increased excitability. The chicken NMc contains multiple cell types with differential expressions of calretinin and parvalbumin, providing a useful model for studying expression mechanisms and specific functions of these calcium-binding proteins.

### CCK in auditory processing and neuronal plasticity

Another important discovery of this study is the characterized CCK expression of NMc2 neurons in chickens. In rat brains, CCK acts as an excitatory neurotransmitter or neuromodulator that can enhance the intrinsic excitability of neurons by either decreasing a cell’s permeability to potassium or enhancing a nonselective cation current (Miller et al., 1997; Deng and Lei, 2006; Chung and Moore, 2007, 2009a, b). This function of CCK is consistent with increased intrinsic excitability of CCK-expressing NMc2 neurons compared with CCK-negative neurons in NMc1 and the more rostral portion of NM. Intriguingly, most CCK-expressing neurons in mammals are thought to be GABAergic (Fallon et al., 1983; Somogyi et al., 1984; Seroogy et al., 1988; Doetsch et al., 1993; Kubota and Kawaguchi, 1997; Kawaguchi and Kubota, 1998). In birds, however, most CCK-expressing neurons are glutamatergic, as evident by coexpression of the glutamatergic cell marker VGLUT2 in chicks (Maekawa et al., 2007) and the lack of colocalization with the GABAergic cell marker GAD65 in the zebra finch (Lovell and Mello, 2011). Few GABAergic neurons were reported in the avian NM (Carr et al., 1989; Code et al., 1989), further supporting the notion that CCK-expressing neurons in NMc2 are non-GABAergic. Together, these studies suggest that CCK may act similarly on modulating cellular physiology across various neuronal types in mammalian and avian brains.

One possible function of CCK-enhanced neuronal excitability may be related to some aspects of neuronal plasticity or integration of multisensory inputs. Local infusion of CCK in the rat auditory cortex potentiates synaptic strength and neuronal responses to auditory stimuli (Li et al., 2014). Interestingly, this plasticity can enable a novel response of these auditory neurons to a visual stimulus after pairing the visual stimulus with a strong auditory stimulus in the presence of CCK (Li et al., 2014). This finding is particularly interesting in light of the distribution of CCK-expressing neurons in the secondary nonlemniscal auditory pathway that is involved in polysensory integration, temporal pattern recognition, and certain forms of learning (Hu, 2003; Lee, 2015). Within this pathway, CCK is strongly expressed in the external nucleus of the inferior colliculus (ICx) and thalamic neurons surrounding the medial geniculate body (MGB; Fallon and Seroogy, 1984; Paloff et al., 1996), as well as the avian counterparts (Ball et al., 1988; Lovell and Mello, 2011). In contrast, neurons in the primary lemniscus auditory pathway including the central nucleus of IC (ICc) and MGB do not express CCK, emphasizing specialized function of CCK in sensory processing. Behaviorally, CCK has been proposed to play important roles in visual imprinting in chickens (Maekawa et al., 2007; Nakamori et al., 2013) and probably certain aspects of song processing in zebra finches (Lovell and Mello, 2011). Our observation that CCK is expressed in the chicken NMc2 but not the remaining NM suggests that NMc2 may have additional function other than representing the very low frequency of the tonotopic axis.

### Potential mechanisms underlying increased excitability of NMc1 and NMc2 neurons

A characterized intrinsic property of NMc1 and NMc2 neurons is increased excitability compared with adendritic NM neurons. Consistent with previous studies (Fukui and Ohmori, 2004), we found that the majority of NMc1 and NMc2 neurons fire multiple APs in response to relatively weak levels of current injection. We further found that a high percentage of NMc1 and NMc2 neurons (>67%) show repetitive firing during sustained current injections. A lower percentage (10%) was reported in Fukui and Ohmori (2004), in which neuron sampling did not include the most caudolateral NM where the major body of NMc2 was located (see their Fig. 2). This discrepancy suggests that NMc2 neurons may be more excitable than NMc1 neurons. Alternatively, age differences should be taken into consideration (E20–E21 in the current study vs. hatchling in Fukui and Ohmori [2004]).

Voltage-dependent potassium channels, in particular the low-voltage activated K_V_1 subfamily (Johnston et al., 2010), may be one mechanism that accounts for the higher excitability of NMc1/NMc2 neurons. Compared with the adendritic NM neurons, which have large amounts of K_V_1 conductances, the caudolateral NM neurons has a lower level of K_V_1.1 mRNA staining (Fukui and Ohmori, 2004). In addition, when K_V_1.1 conductances are blocked, adendritic NM neurons display multiple spikes, resembling the properties of NMc1/NMc2 neurons (Reyes et al., 1994; Rathouz and Trussell, 1998; Hong et al., 2016).

In addition to increased excitability, we found that NMc1/NMc2 neurons generate slower and less reliable APs in a heterogeneous manner. In contrast, AP properties of adendritic NM neurons are highly homogeneous, showing faster and highly reliable APs (Hong et al., 2016). The AP fall rate of NMc1/NMc2 neurons is significantly lower than adendritic NM neurons (Hong et al., 2016). It is well known that high-voltage activated K_V_3 channels are critical regulators of AP kinetics in the repolarizing phase (Johnston et al., 2010). Blockade of K_V_3 channels in adendritic NM neurons leads to slower AP generation (Hong et al., 2016). The caudolateral NM expresses weaker K_V_3 channel expression than other NM regions (Parameshwaran et al., 2001), suggesting lower levels of K_V_3 conductances in NMc1/NMc2 neurons, resulting in their slower AP kinetics.

## Conclusion

The caudolateral NM neurons at the low-frequency end of the tonotopic axis differ from neurons encoding higher frequencies in structure, molecular signaling, and physiology. In addition, the low-frequency NM itself is heterogeneous, containing morphologically and potentially functionally distinct neuron types. These results indicate highly specialized and intricate neuronal mechanisms for processing low-frequency sounds. Further studies aim to characterize these mechanisms and investigate their contribution to auditory temporal processing and binaural hearing.

## Synthesis

Reviewing Editor: Darcy Kelley, Columbia University Decisions are customarily a result of the Reviewing Editor and the peer reviewers coming together and discussing their recommendations until a consensus is reached. When revisions are invited, a fact-based synthesis statement explaining their decision and outlining what is needed to prepare a revision will be listed below. The following reviewer(s) agreed to reveal their identity: Ruili Xie, Felix Felmy.

While the reviewers agree that the paper provides valuable data, they identified a number of concerns that should be addressed:

Title: The title needs revision as the manuscript does not answer which neuronal mechanism is important for low frequency hearing. The manuscript does nicely illustrates the anatomical and electrophysiological differences between the subparts of the NM.

The following concerns should also be addressed:

1. Line 60: “infrasound”: may not be a good term to use here, assuming this refers to human (<20Hz). Chicken should not be sensitive to sounds that they can't hear.

2. Line 79: How is infrasound separated from low frequency sounds?

3. Line 81: This sentence requires a reference regarding the AVCN or rewriting.

4. Line 100: Temporal coding and precision is crucial in the auditory system and it is always nice to state it. However, here in this manuscript no data regarding this topic is presented. Therefore, the authors have to use a different motivation.

5. Line 108: I disagree with the sentence “These results...” I do not see how these results add to the mechanistic understanding how temporal precision is generated and promotes processing of low frequency sounds. This sentence needs to be revised.

6. Line 296: It is unclear why the authors have recorded at room temperature? Is this the standard in the field of auditory physiology of the chicken?

7. Line 422: Major comment: I disagree that CCK is a good biomarker for NMc2, as quite a substantial fraction of NMc1 cells are also labelled. This section needs quantification similarly to the calcium binding proteins. Otherwise this conclusion cannot be drawn.

8. Line 493/496: “calretinin positive ratio“ is misleading as the values are given in percentage. Same holds for parvalbumin positive ratio.

9. Line 516: “Neuron location....” Should be “Neuronal location...”

10. Line 520 and following: Major comment: Here the authors argue that the capacitance is a good indication to segregate cell size. However without biomarkers they were unable to distinguish between NMc1 and NMc2, despite the large difference in dendritic tree. For the data presentation it ise important to indicate the estimated capacitance in respect to the recording position.Furthermore, if capacitance is such a good indication it would be better to show a distribution profile, so the reader can see at least the expected bimodal distribution between NM and NMcs. A table does not help so much. This section requires substantial revision

11. In addition, the authors are not careful with their electrophysiological data. For example, RMP data from this study (junction potential not corrected) were directly compared with their previous study in Hong et al, in which the junction potential was corrected by -10 mV. Of course, they would see a significant difference. (Line530-532; Table 2: RMP data).

12. Line 611: Major comment: The authors need not to speculate but to analyze the correlation between single AP firing neuron and input resistance. A graph that correlates the AP frequency with input resistance is required. The authors only correlate their data to capacitance; here it will help correlate it to input resistance. If this is not feasible the authors need to explain why.

13. Line 632: Major comment: The section about the spontaneous activity is not helpful. First it does not give more insights into the cellular distinctions. Second this observation lacks controls, such as making sure that the patch was stable by pulling out side out patches at the end of a recording. A change in holding potential or blockers would be crucial to substantiate this finding. It would be required to remove this section and work on it some more. This suggestion is in line with the authors last result paragraph where they argue that this is also work in progress.

14. Line 1979: this citation is not referenced in the text.

Figures: Figures regarding the correlation: Remove the NMc1 to NMc2 arrow along the capacitance axis if you were unable to segregate these cells electrophysiologically and when no position within the slice is given. This is misleading and is not represented in the data.

The large variation of the RMP (-50.55 +/- 9.74, n = 30) also suggests that these neurons could be under different health conditions during the recordings, which may lead to the observed variation in physiological properties. A comparison of RMP across different neuronal groups (Fig 10, 11, and 12) could reveal if neurons behave differently under different health conditions at different RMP.

In their last figure the authors nicely show a scheme about their findings. However, by introducing calcium binding proteins they claim that fast spiking neurons require strong calcium buffering to maintain cell integrity. The authors find that neurons that spike more in the slice express rather parvalbumin which is a slow calcium buffer. The authors argue that the lack of calretinin might render these neurons into multi-spiking neurons. However it is not discussed why the more excitable cells have only a slow calcium buffer, or do NMc neurons also express calbindin? Also it is unclear if the steady state current injection is suited to indicate the higher spike rates in vivo, where sound driven input might be mediating different temporal patterns of voltage excursions such as short EPSPs. Since there are several uncertainties it is important to revise either the discussion or the motivation regarding the calcium binding proteins.
